# A bHLH transcription factor, SlbHLH96, promotes drought tolerance in tomato

**DOI:** 10.1093/hr/uhac198

**Published:** 2022-09-06

**Authors:** Yunfei Liang, Fang Ma, Boyu Li, Cong Guo, Tixu Hu, Mingke Zhang, Yan Liang, Jianhua Zhu, Xiangqiang Zhan

**Affiliations:** State Key Laboratory of Crop Stress Biology for Arid Areas and College of Horticulture, Northwest A&F University, Yangling 712100, China; State Key Laboratory of Crop Stress Biology for Arid Areas and College of Horticulture, Northwest A&F University, Yangling 712100, China; State Key Laboratory of Crop Stress Biology for Arid Areas and College of Horticulture, Northwest A&F University, Yangling 712100, China; State Key Laboratory of Crop Stress Biology for Arid Areas and College of Horticulture, Northwest A&F University, Yangling 712100, China; State Key Laboratory of Crop Stress Biology for Arid Areas and College of Horticulture, Northwest A&F University, Yangling 712100, China; State Key Laboratory of Crop Stress Biology for Arid Areas and College of Horticulture, Northwest A&F University, Yangling 712100, China; State Key Laboratory of Crop Stress Biology for Arid Areas and College of Horticulture, Northwest A&F University, Yangling 712100, China; Department of Plant Science and Landscape Architecture, University of Maryland, College Park, MD 20742, USA; School of Life Sciences, Anhui Agricultural University, Hefei 230036, Anhui, China; State Key Laboratory of Crop Stress Biology for Arid Areas and College of Horticulture, Northwest A&F University, Yangling 712100, China

## Abstract

Drought stress caused by water deficit reduces plant productivity in many regions of the world. In plants, basic helix–loop–helix (bHLH) transcription factors regulate a wide range of cellular activities related to growth, development and stress response; however, the role of tomato SlbHLHs in drought stress responses remains elusive. Here, we used reverse genetics approaches to reveal the function of *SlbHLH96*, which is induced by drought and abscisic acid (ABA) treatment. We found that *SlbHLH96* functions as a positive regulator of drought tolerance in tomato. Overexpression of *SlbHLH96* in tomato improves drought tolerance by stimulating the expression of genes encoding antioxidants, ABA signaling molecules and stress-related proteins. In contrast, silencing of *SlbHLH96* in tomato reduces drought tolerance. SlbHLH96 physically interacts with an ethylene-responsive factor, SlERF4, and silencing of *SlERF4* in tomato also decreases drought tolerance. Furthermore, SlbHLH96 can repress the expression of the ABA catabolic gene, *SlCYP707A2*, through direct binding to its promoter. Our results uncover a novel mechanism of *SlbHLH96-*mediated drought tolerance in tomato plants, which can be exploited for breeding drought-resilient crops.

## Introduction

Plant productivity is significantly limited by various environmental challenges, especially drought stress and soil salinity [1]. Drought is one of the most detrimental abiotic stress conditions for plant growth and development, and severely threatens sustainability in agriculture [2]. Drought influences many aspects of plant physiology and causes abnormal changes in cellular processes [3]. In particular, drought stress causes injuries to biological membranes, which significantly elevate ion leakage from plant cells [4–6]. Drought stress also induces the accumulation of excessive reactive oxygen species (ROS), which can cause oxidative damage [7]. Nonetheless, the ROS H_2_O_2_ also acts as a signaling molecule and is involved in regulating stomatal closure, activities of ion channels, and specific stress responses [8]. Drought stress induces the biosynthesis and signaling of the phytohormone abscisic acid (ABA), which triggers a variety of adaptive responses in plants [9]. Under stress conditions, ABA increases the activity of enzymes such as superoxide dismutase (SOD), peroxidase (POD), catalase (CAT), which function in ROS scavenging [10]. In the ABA biosynthetic pathway, the *9-cisepoxycarotenoid dioxygenase* (*NCED*) genes encode key enzymes involved in the speed-limiting step of ABA biosynthesis [11, 12]. So far, three *NCED* genes have been isolated and analyzed in tomato [13–15]. For ABA catabolism, the *CYP707A1*, *A2*, *A3*, and *A4* genes, encoding 8'-hydroxylases, play a pivotal role in ABA oxidation [16–18]. The principal ABA signaling pathway consists of the primary ABA receptor proteins, such as PYR/PYL/RCAR, protein phosphatases of type 2C (PP2Cs) from group A, and SNF1-related kinase 2 (SnRK2) [19–22].

The basic helix–loop–helix (bHLH) family is the second largest family of transcription factors in plants. Members of the bHLH transcription factor family contain two highly conserved and functionally different domains, such as the basic domain and the HLH domain. The basic domain, which is located at the N-terminal end of the bHLH structure, is responsible for binding to an E-box sequence present in the promoter regions of the target genes. The HLH domain, which is located at the C-terminal end of the bHLH structure, is important for protein–protein interactions. These protein complexes work at the E-box region to regulate their target genes’ transcriptional activity to control a variety of developmental processes. Plant bHLH transcription factors are involved in a wide range of cellular activities related to plant growth and development. For example, the bHLH members regulate seed germination [23], flowering time [24], fruit ripening [25], trichome formation [26], and root hair formation [27]. Furthermore, bHLH transcription factors have a role in plant responses to abiotic stressors such as drought, salt, and cold. Drought, salt, and osmotic stress responses are positively regulated by *Arabidopsis AtbHLH122*. Knockout of *AtbHLH122* leads to increased sensitivity to salt and osmotic stress, whereas overexpression of *AtbHLH122* improves plant performance under drought, salt, or osmotic stress conditions [28]. Drought tolerance is improved by overexpression of *OsbHLH148* in rice plants. The possible mechanism is interaction of OsJAZ1 with OsbHLH148 to activate the jasmonate signaling pathway [29]. In *Arabidopsis* and cucumber seedlings, overexpression of *CsbHLH041* improves salt and ABA tolerance [30]. Likewise, overexpression of *SlbHLH22* increases drought and salt tolerance in tomato [31]. Ectopic expression of maize *ZmbHLH55* in *Arabidopsis* improves salt stress tolerance, which is associated with higher ascorbic acid levels in the transgenic plants [32]. In apple, MdbHLH3 improves cold resistance by elevating anthocyanin accumulation via transcriptional regulation of the anthocyanin biosynthetic genes *MdDFR* and *MdUFGT* under cold conditions [33]. Furthermore, PtrbHLH regulates *PtrCAT* expression by direct binding to its promoter and overexpression of *PtrbHLH* in transgenic pummelo (*Citrus grandis*) improves cold tolerance [34].

Ethylene-responsive factors (ERFs) contain an AP2 DNA-binding domain, and this protein family is widely found in higher plants but is absent in mammals, fungi, and yeast [35–38]. Members of the ERF protein family are shown to play key roles in many abiotic stress responses in plants. For example, overexpression of the tomato ERF transcription factor *SlTSRF1* in rice improves drought tolerance by upregulating the expression of stress-responsive genes [39]. In addition, overexpression of *OsERF19* in rice plants enhances resistance to salt stress while causing an ABA hypersensitivity phenotype [40]. Overexpression of *OsERF115* improves heat tolerance in rice plants at the vegetative stage [41]. Furthermore, overexpression of *PagERF16* increases salt sensitivity in poplar [42]. In *Arabidopsis*, heterologous overexpression of *SlERF84* increases drought and salt stress resistance [43]. Overexpression of *SlERF5* in tomato plants shows similar effects [44].

Tomato (*Solanum lycopersicum* L.) is one of the world’s most commonly grown and commercially significant vegetable crops [45]. Tomato growth, development, and productivity are severely affected by various abiotic stresses, such as salinity, drought, chilling, and high temperatures [46]. Therefore, improving abiotic stress tolerance is increasingly vital for sustainable tomato production. In this study, we used multiple genetics approaches and revealed that *SlbHLH96* is vital for drought tolerance in tomato plants. Our results show that overexpression of *SlbHLH96* in tomato improves drought tolerance, whereas silencing of *SlbHLH96* in tomato reduces drought tolerance. Furthermore, we showed that SlbHLH96 physically interacts with SlERF4, and silencing of *SlERF4* in tomato decreases drought tolerance. SlbHLH96 binds to the promoter of *SlCYP707A2* to downregulate its expression to fine-tune the expression of ABA response-related genes.

## Results

### Identification and characterization of *SlbHLH96* gene in tomato

From RNA-seq experiments (accession numbers SAMN14996375–14996413), we found that *SlbHLH96* is upregulated by drought treatment in tomato (Supplementary Data Figs S1 and S2A), suggesting its potential roles in drought stress responses. *SlbHLH96* encodes a protein with 441 amino acid residues having a molecular weight of 48.74 kDa. The theoretical isoelectric point (pI) of this protein is 6.54, with an instability index of 53.40 and an aliphatic index of 64.81. Conserved domain analysis showed that SlbHLH96 possesses the typical structure of the bHLH transcription factors. Phylogenetic analysis suggested that SlbHLH96 is closely related to potato StbHLH117 (Supplementary Data Fig. S2B). *SlbHLH96* was highly expressed in leaf and flower tissues while its expression was relatively low in root and fruit tissues (Supplementary Data Fig. S3). To investigate the subcellular localization of SlbHLH96, we transiently expressed the SlbHLH96–GFP fusion protein in tobacco leaves. Our results showed that GFP protein driven by the 35S promoter spread throughout the cell, whereas the SlbHLH96–GFP fusion protein was only observed in the nucleus (Supplementary Data Fig. S4).

### *SlbHLH96* expression is responsive to multiple abiotic stresses and hormone treatments

**Figure 1:**
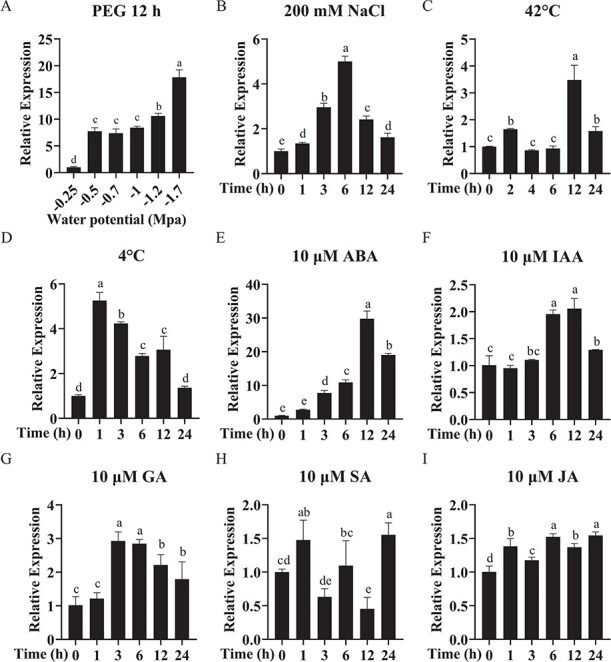
*SlbHLH96* is responsive to multiple abiotic stress and hormone treatments in tomato plants. (A–I) *SlbHLH96* expression in tomato seedlings after treatment with low water potential conditions created through PEG-infused agar medium, salt stress (NaCl), heat stress (42°C), cold stress (4°C), ABA, IAA, GA, SA, and JA. The data are means ± standard deviation (*n* = 3). Letters indicate significant differences according to one-way ANOVA (Tukey’s test; *P* < .05).

We examined the expression profile of *SlbHLH96* under different abiotic stress and hormone treatments. *SlbHLH96* expression was substantially induced by low water potential treatments imposed by infusion of polyethylene glycol (PEG; average molecular weight 8000) in the growth medium, and this is consistent with our RNA-seq results from drought-treated plants grown in soil (Fig. 1A and Supplementary Data Fig. S2A). Similar expression patterns of *SlbHLH96* were observed after ABA treatment (Fig. 1E). These results suggest that *SlbHLH96* may function in drought stress responses in an ABA-dependent manner. The expression of *SlbHLH96* appeared to be responsive to other abiotic stresses or hormones (Fig. 1B–D and F–I). However, its expression levels under these conditions were much lower compared with those under drought or PEG treatment. These results indicate that *SlbHLH96* may play a major role in drought stress responses through an ABA-dependent pathway.

### Overexpression of *SlbHLH96* in tomato improves drought tolerance

**Figure 2:**
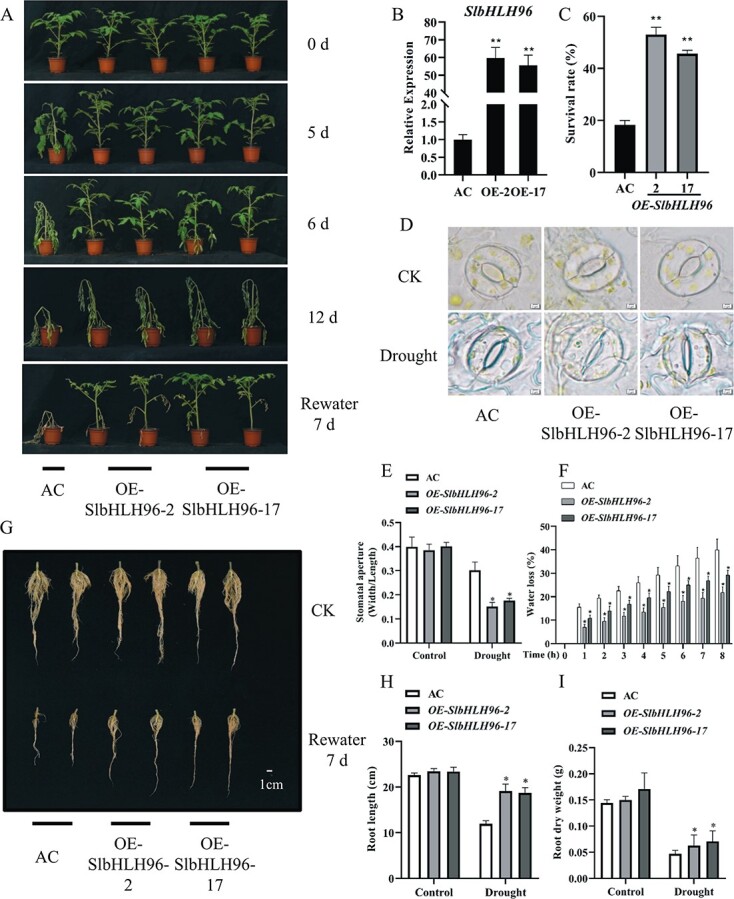
Overexpression of *SlbHLH96* in tomato improves drought tolerance. (A) Morphology and responses of wild-type (AC) and *SlbHLH96* overexpression plants. (B) Expression of *SlbHLH96* in AC and *SlbHLH96* overexpression plants. (C) Survival rates of plants shown in (A) after a recovery for 7 days. (D, E) Stomatal aperture analysis. Scale bar in (D) = 2 μm. (F) Water loss from detached leaves of AC and *SlbHLH96* overexpression plants. (G) Root morphology of AC and *SlbHLH96* overexpression plants. (H) Quantification of root length of plants shown in (G). (I) Quantification of root dry weight of plants shown in (G). Data are means ± standard deviation [*n* = 3 (there were at least 10 plants per biological replicate)]. Significant differences in mean values are indicated by asterisks: ^*^P < .05, ^**^P < .01 (Student’s *t*-test).

To investigate the biological significance of *SlbHLH96* in drought tolerance, we produced tomato plants overexpressing *SlbHLH96* in the ‘Ailsa Craig’ (AC) genetic background (wild type). The expression levels of *SlbHLH96* in two independent *T*_2_ homozygous transgenic lines were examined by qRT–PCR analysis and the results revealed that the transcript abundance of *SlbHLH96* in the OE-SlbHLH96-2 and OE-SlbHLH96-17 plants was ~60-fold and 55-fold that of the AC plants, respectively (Fig. 2B). We then examined the drought tolerance of the 30-day-old soil-grown *SlbHLH96* overexpression lines and AC plants. Both genotypes were subjected to continuous drought treatment for 12 days. At the beginning of the experiment, the overexpression plants showed a phenotype similar to that of the AC plants (Fig. 2A). However, after 5 days of drought the AC plants started to display a leaf wilting phenotype while the *SlbHLH96* overexpression plants were essentially healthy. Although both genotypes became wilted at the end of 12 days of drought treatment, it was obvious that the AC plants displayed more severe drought-induced damage (such as leaves with drooping petioles) than the overexpression plants (Fig. 2A). All the plants were then re-irrigated for recovery. After recovery for 7 days, ~45–53% of the wilted *SlbHLH96* overexpression plants survived, whereas <20% of the wilted AC plants survived (Fig. 2A and C). In addition, the *SlbHLH96* overexpression plants developed more vigorous root systems than the AC plants during the drought and the recovery period (Fig. 2G–I). We also examined stomatal aperture to determine whether the improved drought stress tolerance in the *SlbHLH96* overexpression plants is related to the difference in stomatal movement. We found that the *SlbHLH96* overexpression plants had much narrower stomatal apertures than the AC plants under drought stress (Fig. 2D and E). Consistent with this observation, detached leaves from the *SlbHLH96* overexpression plants showed a slower water loss rate than leaves from the AC plants (Fig. 2F). These findings indicate that overexpression of *SlbHLH96* in tomato improves drought tolerance at least partly by minimizing water loss.

### *SlbHLH96* is essential for ROS detoxification under drought stress

**Figure 3:**
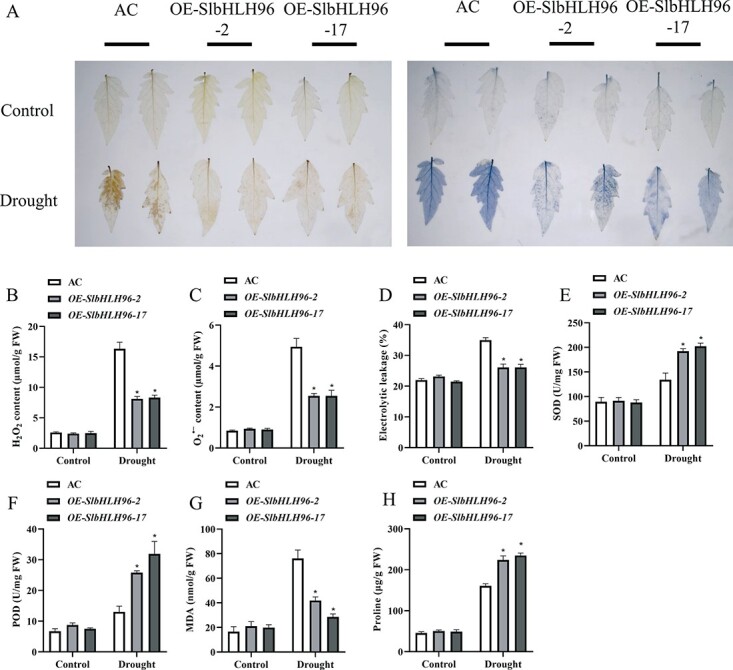
*SlbHLH96* overexpression plants sowed less stress-induced damage than AC plants. (A) DAB staining for H_2_O_2_ and NBT staining for superoxide. (B) H_2_O_2_ content. (C) O_2_^⋅−^ content. (D) Electrolyte leakage assay. (E) SOD activity. (F) POD activity. (G) MDA content. (H) Proline content. Data are means ± standard deviation [*n* = 3 (there were at least 10 plants per biological replicate)]. Significant differences in mean values are indicated by an asterisk: ^*^P < .05 (Student’s *t*-test).

O_2_^⋅−^ and H_2_O_2_ are the two prominent ROS molecules that are commonly accumulated under abiotic stress. Thus, we detected the accumulation of O_2_^⋅−^ and H_2_O_2_ in the AC and *SlbHLH96* overexpression plants through nitro blue tetrazolium (NBT) staining (for O_2_^⋅−^) and 3,3'-diaminobenzidine (DAB) staining (for H_2_O_2_) methods under control and drought conditions. Under control conditions, there were no detectable differences in the accumulations of O_2_^⋅−^ and H_2_O_2_ between the AC and the *SlbHLH96* overexpression plants. In contrast, under drought stress, the accumulations of O_2_^⋅−^ and H_2_O_2_ in the leaves of the *SlbHLH96* overexpression plants were substantially lower than those in the leaves of the AC plants (Fig. 3A–C). These results suggest that *SlbHLH96* overexpression plants possess an enhanced ROS-scavenging capacity under drought stress. Consistent with this observation, we found that the *SlbHLH96* overexpression plants showed increased activities of antioxidant enzymes such as SOD and POD, and elevated proline accumulation, and reduced membrane damage (indicated by reduced electrolyte leakage) and less malondialdehyde (MDA) content under drought stress (Fig. 3D–H). No significant differences in these physiological and biochemical parameters were detected between the AC and *SlbHLH96* overexpression plants under control conditions. Taken together, these results indicated that the *SlbHLH96* overexpression plants suffered less stress-induced damage than the AC plants.

### *SlbHLH96* regulates the expression of genes involved in ABA biosynthesis, catabolism, and signal transduction

**Figure 4:**
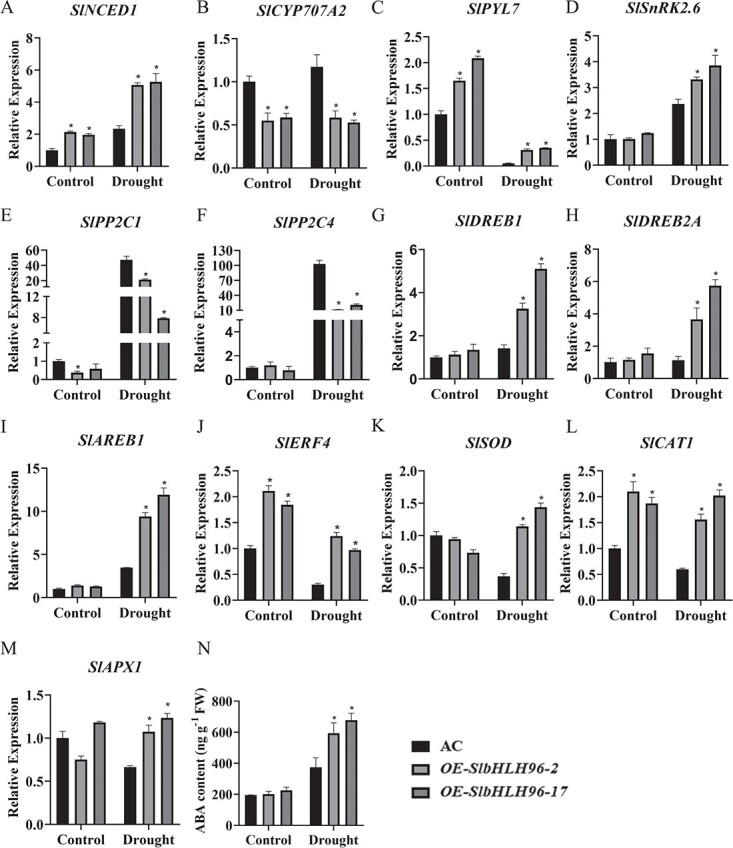
Expression profiles of a set of ABA-related genes and stress-related genes as influenced by the *SlbHLH96* overexpression in tomato plants. Relative expression of (A) ABA biosynthetic gene-*SlNCED1*, (B) ABA catabolism gene-*SlCYP707A2*, (C–F) ABA signal transduction-related genes, and (G–M) stress-related genes. (N) ABA levels in *SlBHLH96* overexpression plants. Data are means ± standard deviation (*n* = 3). Significant differences in mean values are indicated by an asterisk: ^*^P < .05 (Student’s *t*-test).

The increased expression level of *SlbHLH96* under ABA treatment prompted us to examine whether the expression of genes involved in ABA biosynthesis, catabolism, and downstream signal transduction pathway was altered in the *SlbHLH96* overexpression plants under drought conditions. The expression of *SlNCED1*, which encodes a key enzyme in ABA biosynthesis, increased in the *SlbHLH96* overexpression plants under both control and drought conditions (Fig. 4A), whereas the expression of *SlCYP707A2*, which encodes a major ABA 8'-hydroxylase essential for ABA catabolism, decreased in the *SlbHLH96* overexpression plants under both control and drought conditions (Fig. 4B). In addition, we showed that the expression of one of the ABA receptors, *SlPYL7*, increased in the *SlbHLH96* overexpression plants under both control and drought conditions while a substantial reduction in the expression of *SlPP2C1* was found in the *SlbHLH96* overexpression plants under both control and drought conditions (Fig. 4C and E). Furthermore, the expression of *SlPP2C4* decreased and the expression of *SlSnRK2.6* increased in the *SlbHLH96* overexpression plants under drought conditions (Fig. 4D and F). In addition to the above changes, the reduced expression level of *SlCYP707A2* in the SlbHLH96 overexpression plants suggests that SlbHLH96 might act as a negative regulator for ABA catabolism. We then analyzed ABA levels using LC–MS/MS in the *SlbHLH96* overexpression and AC plants. We found that ABA levels were much higher in the *SlbHLH96* overexpression plants than in the AC plants under drought stress (Fig. 4N), and a higher ABA content usually resulted in improved drought resistance. The results suggest that altered expression of genes involved in ABA biosynthesis, ABA catabolism, and ABA signaling may contribute to the increased drought tolerance of the *SlbHLH96* overexpression plants.

### Expression profiles of stress-related genes in *SlbHLH96* overexpression plants under drought stress

To uncover the potential molecular mechanisms underlying the improved tolerance of the *SlbHLH96* overexpression plants to drought stress, we investigated the transcript levels of stress-related genes, including *SlDREB1*, *SlDREB2A*, *SlAREB1*, *SlSOD*, *SlCAT1*, and *SlAPX1*. Compared with the AC plants, the *SlbHLH96* overexpression plants showed higher expression levels of *SlDREB1*, *SlDREB2A*, and *SlAREB1* under drought treatment, whereas no obvious differences in the expression of these genes were detected under control condition (Fig. 4G–I). The expression levels of genes encoding antioxidant enzymes such as *SlSOD*, *SlCAT1*, and *SlAPX1* were significantly higher in the *SlbHLH96* overexpression plants than in the AC plants under drought (Fig. 4K–M). These findings indicate that *SlbHLH96*-mediated improved drought tolerance is associated with the expression of stress-related genes.

### Silencing of *SlbHLH96* in tomato reduces drought tolerance

**Figure 5:**
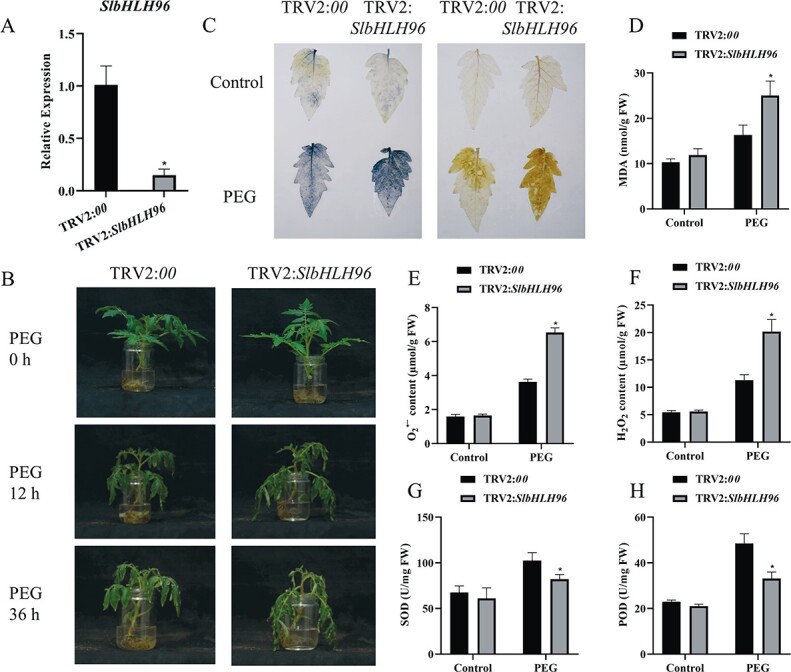
Silencing of *SlbHLH96* in tomato reduces drought stress tolerance. (A) Expression of *SlbHLH96* in *SlbHLH96*-silenced (*TRV2:SlbHLH96*) and control (*TRV2:00*) plants. (B) Phenotype of *SlbHLH96*-silenced and control plants exposed to 15% PEG8000. (C) NBT staining for superoxide and DAB staining for H_2_O_2_. (D) MDA content. (E) O_2_^⋅−^ content. (F) H_2_O_2_ content. (G) SOD activity. (H) POD activity. Data are means ± standard deviation [*n* = 3 (there were at least 10 plants per biological replicate)]. Significant differences in mean values are indicated by an asterisk: ^*^P < .05 (Student’s *t*-test).

To further reveal the essentiality of *SlbHLH96* in basal drought tolerance, the expression of *SlbHLH96* was suppressed by virus-induced gene silencing (VIGS) in tomato. We observed that *SlPDS*-silenced plants showed a photo-bleached phenomenon (Supplementary Data Fig. S5). The expression of *SlbHLH96* in the *TRV2:SlbHLH96* plants significantly decreased by 85% (Fig. 5A), indicating that *SlbHLH96* was efficiently silenced. The control (*TRV2:00*) and *TRV2:SlbHLH96* plants were immersed in 15% PEG8000 to simulate drought stress. The *TRV2:SlbHLH96* plants became wilted sooner than the *TRV2:00* plants after the PEG treatment (Fig. 5B). Under drought stress, the *TRV2*:*SlbHLH96* plants showed a higher MDA content than the *TRV2:00* plants (Fig. 5D). ROS assay results showed that the accumulations of O_2_^⋅−^ and H_2_O_2_ were higher in the *TRV2:SlbHLH96* plants under drought stress (Fig. 5C, E, and F). Furthermore, we measured the activities of SOD and POD and found that their activities were substantially decreased in the *TRV2:SlbHLH96* plants under drought stress (Fig. 5G and H). These results indicate that silencing of *SlbHLH96* results in drought sensitivity in tomato plants.

**Figure 6:**
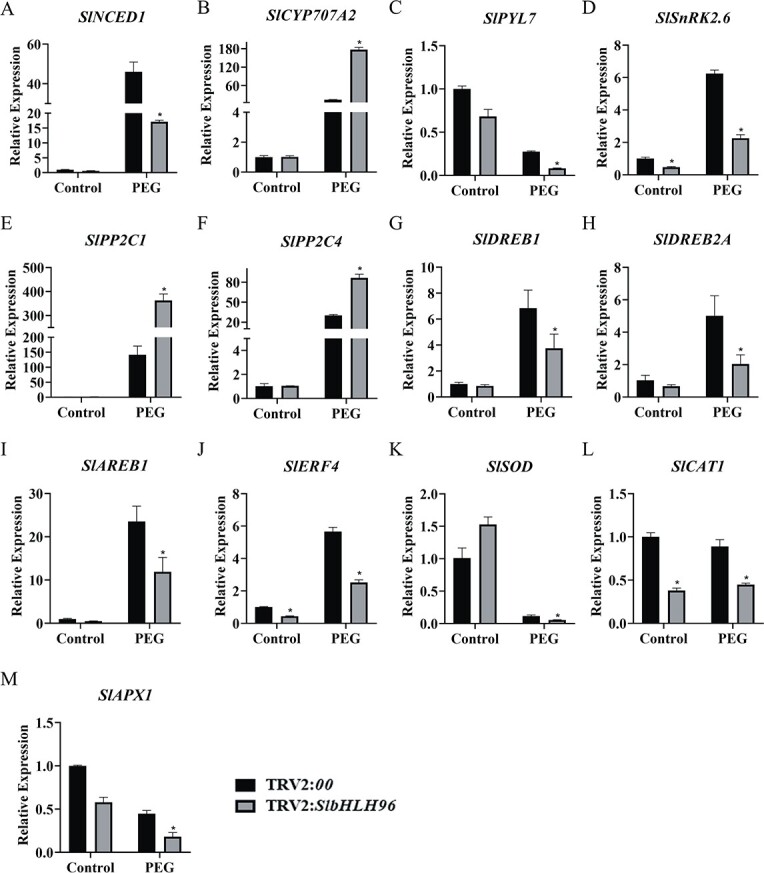
Expression profiles of a set of ABA-related genes and stress-related genes in *SlbHLH96*-silenced plants. (A) ABA biosynthetic gene *SlNCED1*. (B) ABA catabolism gene *SlCYP707A2*. (C–F) ABA signal transduction-related genes. (G–M) Stress-related genes. Data are means ± standard deviation (*n* = 3). Significant differences in mean values are indicated by an asterisk: ^*^P < .05 (Student’s *t*-test).

We subsequently determined the expression levels of genes involved in ABA biosynthesis, catabolism, and signal transduction in the *SlbHLH96*-silenced and *TRV2:00* control plants. The qRT–PCR analysis revealed that *SlNCED1* expression was lower and *SlCYP707A2* expression was significantly higher in the *TRV2:SlbHLH96* plants under drought stress (Fig. 6A and B). In addition, we observed a reduction in the expression of *SlPYL7* and *SlSnRK2.6* in the *TRV2:SlbHLH96* plants under drought stress (Fig. 6C and D). However, upregulated expression of *SlPP2C1* and *SlPP2C4* was detected in the *TRV2:SlbHLH96* plants under drought stress (Fig. 6E and F). Finally, we analyzed the expression of some stress- and antioxidant-related genes and found that their expression levels were lower in the *SlbHLH96*-silenced plants than in *TRV2:00* control plants under drought stress (Fig. 6G–M).

### SlbHLH96 interacts with SlERF4

**Figure 7:**
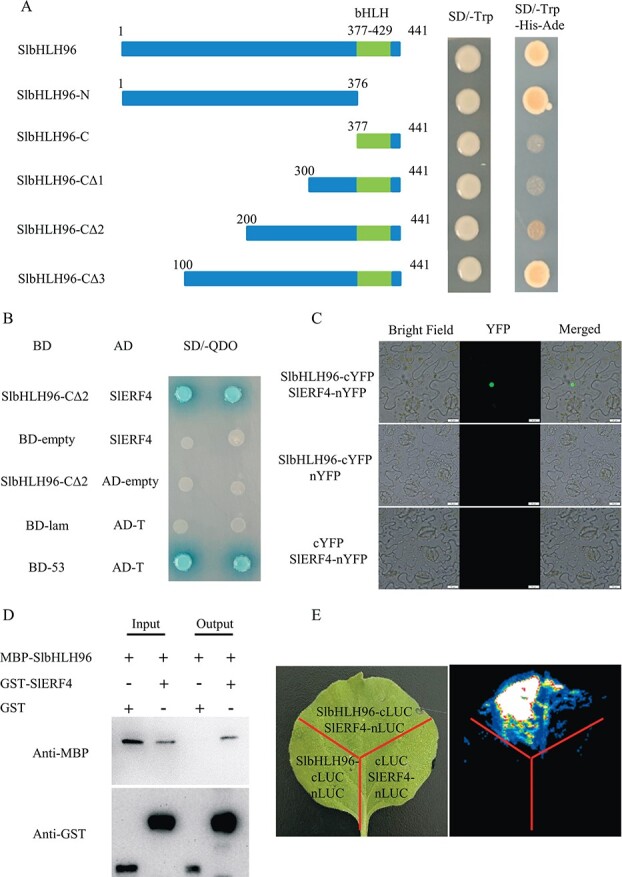
SlbHLH96 and SlERF4 physically interact with each other. (A) Self-activation test of SlbHLH96 protein in yeast. Schematic diagram showing the SlbHLH96 deletions to test self-activation activity. (B) Y2H assays of SlbHLH96 and SlERF4. Yeast cells were grown on SD−Ade−His−Leu−Trp with 20 μg/ml X-α-Gal. (C) BiFC analysis of SlbHLH96 and SlERF4 in tobacco. Scale bar = 20 μm. (D) Pull-down assay of SlbHLH96 and SlERF4 *in vitro*. MBP–SlbHLH96 and GST–SlERF4 proteins were purified and detected by western blotting. (E) Split-luciferase assay of SlbHLH96 and SlERF4 in tobacco.

To identify proteins that interact with SlbHLH96, a bioinformatics prediction was performed using STRING (https://cn.string-db.org/). This *in silico* analysis showed a possibility that SlbHLH96 could interact with SlERF4. *SlERF4* is ubiquitously expressed in all tissues, with slightly less expression in unopened flower buds, fully opened flowers, and ripening fruits at the breaker stage (Supplementary Data Fig. S3). The transcriptional activation activity of SlbHLH96 was evaluated using a GAL4 activation system in yeast. Our results suggest that SlbHLH96 has self-activation activity in yeast, and the C-terminal segments of SlbHLH96 (SlbHLH96-C, SlbHLH96-CΔ1, and SlbHLH96-CΔ2), including the conserved bHLH domain, do not display the self-activation activity (Fig. 7A). It is possible that the amino acid residues at 101–199 from the N-terminal end of SlbHLH96 confer the self-activation activity because the SlbHLH96-CΔ3 segment still has self-activation activity compared with the SlbHLH96-CΔ2 segment. Truncated SlbHLH96 (SlbHLH96-CΔ2) was able to interact with SlERF4 in yeast (Fig. 7B). Bimolecular fluorescence complementation (BiFC) assays were performed to confirm the direct interaction between SlbHLH96 and SlERF4 in tobacco plants. Co-expression of SlbHLH96–cYFP and SlERF4–nYFP generated fluorescent signals in the nucleus, where both these two transcription factors are localized (Fig. 7C). The pull-down assay and split-luciferase assay also confirmed the interaction between full-length SlbHLH96 and SlERF4 (Fig. 7D and E). In addition, the expression levels of *SlERF4* were higher in the *SlbHLH96* overexpression plants than in the AC plants under control conditions and drought treatment (Fig. 4J). In contrast, the expression of *SlERF4* was reduced in the *SlbHLH96*-silenced plants under control conditions and drought treatment (Fig. 6J). These results suggest that *SlbHLH96* may function as a positive regulator for *SlERF4* expression.

### Silencing of *SlERF4* in tomato decreases tolerance to drought stress

**Figure 8:**
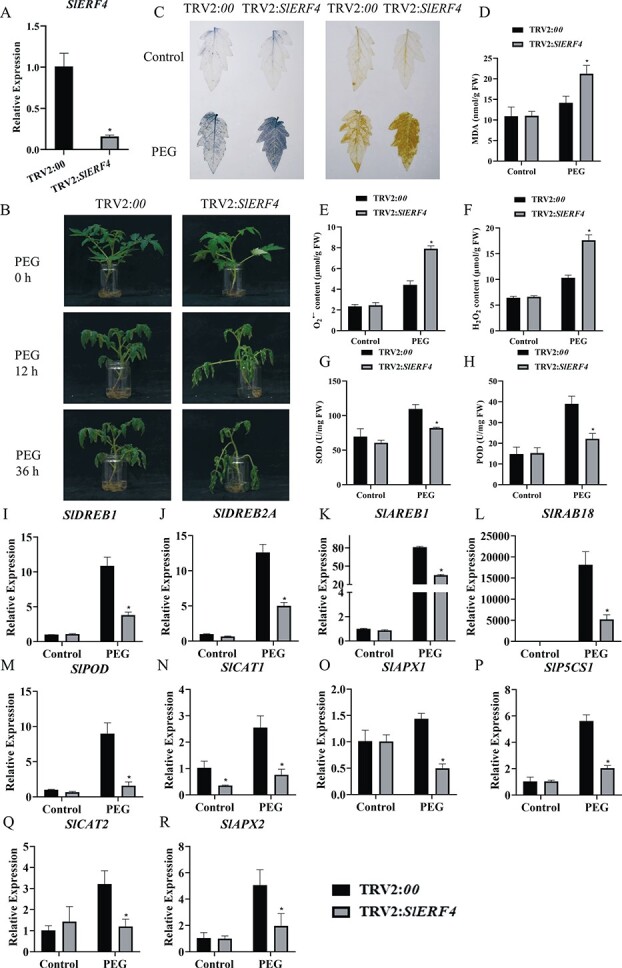
Silencing of *SlERF4* in tomato reduces drought stress tolerance. (A) Expression of *SlERF4* in *SlERF4*-silenced (*TRV2:SlERF4*) and control (*TRV2:00*) plants. (B) Phenotype of *SlERF4*-silenced and control plants exposed to 15% PEG8000. (C) NBT staining for superoxide and DAB staining for H_2_O_2_. (D) MDA content. (E) O_2_^⋅−^ content. (F) H_2_O_2_ content. (G) SOD activity. (H) POD activity. (I–R) Expression profiles of stress-related genes in *SlERF4*-silenced and control plants under drought stress. Data are means ± standard deviation (*n* = 3). Significant differences in mean values are indicated by an asterisk: ^*^P < 0.5 (Student’s *t*-test).

A previous study showed that *SlERF4* antisense plants exhibited salt stress-dependent growth inhibition [47]. However, the function of *SlERF4* in the response to drought stress in tomato remains unknown. A particular 300-bp sequence of *SlERF4* was selected to knock down *SlERF4* following a VIGS protocol. Our qRT–PCR analysis revealed that the expression of *SlERF4* was significantly reduced by VIGS in the *TRV2:SlERF4* tomato plants (Fig. 8A). Compared with the control (*TRV2:00*) plants, *SlERF4* knockdown (*TRV2:SlERF4*) plants were sensitive to drought stress simulated by 15% PEG8000 (Fig. 8B). The *TRV2:SlERF4* plants showed a higher MDA content than the *TRV2:00* plants (Fig. 8D). In addition, ROS assay showed that the *TRV2:SlERF4* plants accumulated more O_2_^⋅−^ and H_2_O_2_ than the *TRV2:00* plants (Fig. 8C, E, and F). Consistent with this observation, SOD and POD activities were lower in the *TRV2:SlERF4* plants (Fig. 8G–H). We subsequently observed that the transcript levels of some stress- and antioxidant-related genes were significantly lower in the *SlERF4* knockdown plants under drought stress (Fig. 8I–R).

### SlbHLH96 can repress *SlCYP707A2* expression through direct binding to *cis*-elements in its promoter

**Figure 9:**
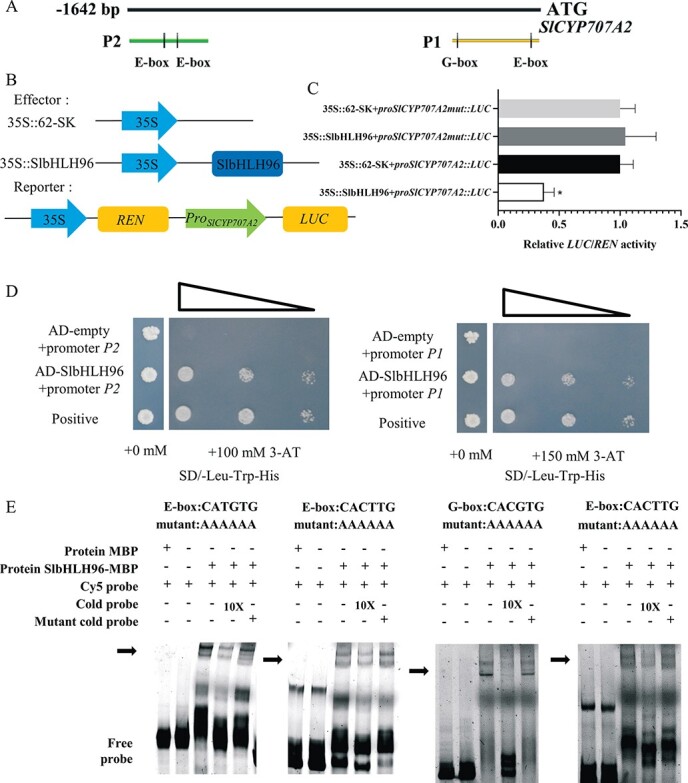
SlbHLH96 directly binds to the *SlCYP707A2* promoter and represses its expression. (A) Schematic diagrams of G-box and E-box motifs in the putative *SlCYP707A2* promoter. (B) Schematic representation of the reporter and effector. (C) Relative luciferase activity from the dual-luciferase reporter assays in *N. benthamiana* leaves. *proSlCYP707A2mut*: three E-boxes and one G-box of the *SlCYP707A2* promoter were mutated. (D) Y1H assay demonstrating that SlbHLH96 binds directly to the *SlCYP707A2* promoter. pGADT7 served as a negative control. (E) Interaction between SlbHLH96 protein and the *SlCYP707A2* promoter in the EMSA. Protein MBP was the negative control. Cold probe was unlabeled. Mutated probes of *SlCYP707A2* promoter fragments had a mutated E-box and G-box where CANNTG was replaced with AAAAAA. The black arrow indicates the specific binding complexes. Data are means ± standard deviation (*n* = 3). Significant differences in mean values are indicated by an asterisk: ^*^P < .05 (Student’s *t*-test).

A previous study showed that AtbHLH122 can bind to the G-box/E-box in the *AtCYP707A3* promoter [28]. SlCYP707A2 was identified as the closest homolog to AtCYP707A3 (75.05% similarity at the amino acid level). *SlCYP707A2* is expressed at a relatively low abundance in all tissues in tomato (Supplementary Data Fig. S3). The consensus *cis*-elements (one G-box and three E-boxes) were found in the putative promoter region of *SlCYP707A2* (Supplementary Data Fig. S6A). *AtCYP707A3* and *SlCYP707A2* share ~40% sequence similarity at the DNA level in their putative promoter regions (Supplementary Data Fig. S6B). To examine whether SlbHLH96 can repress the transcription of *SlCYP707A2*, the dual-luciferase reporter assay was performed in tobacco plants. The dual-luciferase assay revealed that SlbHLH96 can repress the activity of the *SlCYP707A2* promoter. After mutating all three E-boxes and one G-box, SlbHLH96 could not repress the activity of the *SlCYP707A2*-mut promoter (Fig. 9A–C). Furthermore, SlbHLH96 was able to bind to the *SlCYP707A2* promoter fragments that contained the *cis*-elements determined by yeast-one hybrid (Y1H) assays (Fig. 9D). The electrophoretic mobility shift assay (EMSA) further confirmed that SlbHLH96 could directly target the *SlCYP707A2* promoter by binding to the E-box and G-box *cis*-elements (Fig. 9E). The signal was reduced when an unlabeled *SlCYP707A2* probe was introduced to the system as a cold probe (Fig. 9E). Collectively, these results indicate that SlbHLH96 can repress *SlCYP707A2* expression through direct binding to the *cis*-elements in its promoter. Furthermore, we determined whether SlERF4 could regulate the expression of *SlCYP707A2*. The dual-luciferase assay revealed that SlERF4 could not regulate the expression of *SlCYP707A2*, but the interaction between SlERF4 and SlbHLH96 enhanced the inhibitory effect of SlbHLH96 on the expression of *SlCYP707A2* (Supplementary Data Fig. S7). Previous studies showed that bHLH proteins CsbHLH18 and PtrbHLH could bind and regulate antioxidant genes [34, 48, 49], but our results indicated that SlbHLH96 could not regulate the antioxidant enzyme genes *SlCAT1* and *SlPOD* in tomato (Supplementary Data Fig. S8).

## Discussion

In recent years, a significant reduction in crop productivity due to drought stress has emerged as a critical issue for the sustainability of global agriculture. Numerous investigations have demonstrated that overexpression of bHLH transcription factors can generate drought resistance in diverse plant species [28, 29, 31]. Nevertheless, few tomato bHLH proteins have been reported to play vital roles in drought responses. Herein, we characterized a bHLH transcription factor gene, *SlbHLH96*, which is responsive to drought stress and ABA treatment. Overexpression of *SlbHLH96* enhanced drought resistance, while silencing of *SlbHLH96* in tomato reduced drought tolerance, which was associated with ROS metabolism. The AP2/ERF transcription factor family includes DREB proteins as a subfamily. *DREB* genes have been implicated in drought stress responses in a variety of plant species [50–52]. *SlAREB1* is a bZIP transcription factor that belongs to the AREB/ABF subfamily, and it confers drought and salt stress tolerance in tomato [53]. In the current study, the expression of stress-related genes (*SlDREB1*, *SlDREB2A*, and *SlAREB1*) increased significantly in the *SlbHLH96* overexpression plants under drought stress, while the expression of these stress-related genes decreased significantly in the *SlbHLH96*-silenced plants.

ABA is sensed by the PYL ABA receptor proteins [20, 21]. In this study, *SlPYL7* expression increased in the *SlbHLH96* overexpression plants under both control and drought conditions while downregulated expression of *SlPYL7* was detected in the *SlbHLH96*-silenced plants under drought stress. In *Arabidopsis*, AtPYL9 promotes drought resistance and leaf senescence [54]. In comparison with wild-type plants, *SlPYL9* overexpression lines showed increased drought tolerance, but *SlPYL9*-RNAi lines showed weak tolerance [55]. Overexpression of cotton *PYL10*, *PYL12*, and *PYL26* independently in *Arabidopsis* improves tolerance to drought stress [56]. ZmPYL8 or ZmPYL9 overexpression in *Arabidopsis* increases drought resistance [57]. In this study, downregulated expression levels of *SlPP2C1* and *SlPP2C4* were found in the *SlbHLH96* overexpression plants under drought conditions, while upregulated expression levels of *SlPP2C1* and *SlPP2C4* were detected in the *SlbHLH96*-silenced plants under drought stress. *SlPP2C3* overexpression plants were found to be more drought-sensitive than wild-type plants, while *SlPP2C3*-RNAi plants showed a considerable increase in drought tolerance [58]. Compared with wild-type plants, *SlPP2C1*-RNAi transgenic lines showed improved drought tolerance [59]. In the case of ABA signaling, *OsPP2C9* has a positive effect on plant growth but a detrimental effect on drought tolerance [60]. Wheat PP2C-a10 decreased drought tolerance of transgenic *Arabidopsis* [61]. In *Arabidopsis*, overexpression of *ZmPP2C-A6* reduced drought tolerance [62]. In this study, increased *SlSnRK2.6* expression was found mainly under drought stress in the *SlbHLH96* overexpression plants, while downregulated expression of *SlSnRK2.6* was detected in the *SlbHLH96*-silenced plants under drought stress. In transgenic *Arabidopsis*, overexpression of cucumber *CsSnRK2.5* improves drought tolerance [63]. Overexpression of *MpSnRK2.10* confers resistance to drought in apple [64]. Drought tolerance is severely diminished in the *Arabidopsis srk2d*/*e*/*i* triple mutant [65, 66].

A previous bioinformatics prediction showed that SlbHLH96 is a non-G-box-binding protein [67]. Although SlbHLH132 is predicted as a non-DNA-binding protein, the EMSA result showed that SlbHLH132 is a G-box *cis*-element DNA-binding protein [68]. By Y1H, EMSA, and dual-luciferase analyses, we demonstrated that SlbHLH96 directly binds to *cis*-elements (E-box and G-box) in the *SlCYP707A2* promoter region to downregulate its transcription. The increased level of endogenous ABA in the *SlbHLH96* overexpression plants might be caused by the direct repression of *SlCYP707A2* transcription by SlbHLH96. Improved ABA-inducible gene expression and increased drought tolerance are both seen in the *atcyp707a3* mutant [17]. In sweet cherry, when *PacCYP707A1* was silenced, fruits were more resistant to drought stress than control fruits [69].

Multiple functions of tomato SlERF4 have been reported. Compared with the wild type, *SlERF4* knockdown tomato plants displayed a salt stress-sensitive phenotype [47]. SlERF4 is desumoylated by the *Xanthomonas* type III effector XopD, which suppresses ethylene responses and enhances pathogen growth. During Xcv infection, SlERF4 is essential for the activation of XopD-repressed genes [70]. Overexpression of *ERF4-SRDX* (chimeric dominant repressor version) causes a significant delay in ripening as well as increased climacteric ethylene production [71]. SlERF4 regulates the expression of *SlIAA27*, which controls ethylene and auxin signaling [72]. *SlERF5* overexpression in tomato plants led to enhanced salt and drought stress resistance [44]. The evolutionary relationship between SlERF4 and SlERF5 is very close. *SlERF4* has been functionally characterized under salt stress, disease resistance, fruit ripening, and auxin signaling. However, the function of *SlERF4* in drought stress remains unclear. In this study, we demonstrated that SlbHLH96 physically interacts with SlERF4. The *SlERF4* knockdown plants showed a higher MDA content than the control plants. Notably, MDA is a primary indicator of the peroxidation of membrane polyunsaturated fatty acids. Moreover, SOD and POD activities were higher in the control plants. Transcript levels of some stress-related genes and antioxidant-related genes were significantly lower in the *SlERF4* knockdown plants. These results suggest that the SlbHLH96–SlERF4 complex is important in the regulation of expression of genes for ROS scavenging and stress responses under drought through an undefined mechanism.

**Figure 10:**
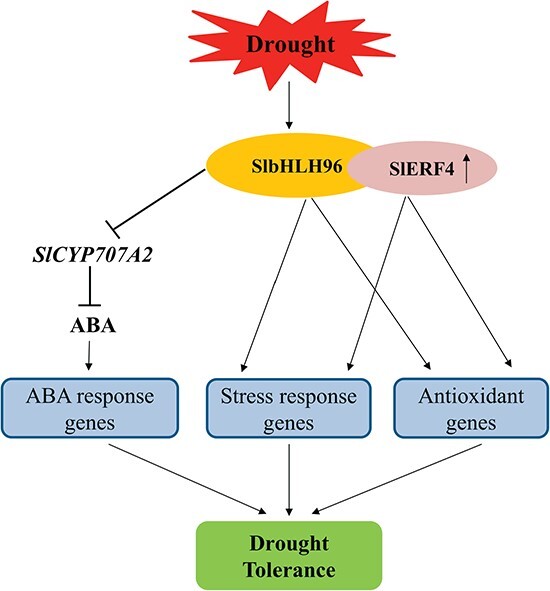
Proposed model for SlbHLH96 function under drought stress in tomato. *SlbHLH96* is induced by drought stress. SlbHLH96 directly binds to *cis*-elements in the *SlCYP707A2* promoter and downregulates its transcription, leading to an increased level of ABA, which, in turn, regulates the expression of ABA response-related genes. Furthermore, SlbHLH96 interacts with SlERF4, and the SlbHLH96–SlERF4 complex may have additive effect on the expression of *SlCYP707A2*. SlbHLH96 and SlERF4 may contribute to drought stress tolerance by modulating the expression of genes encoding antioxidants and stress-related genes.

Based on the results of this study, we proposed a working model for the function of SlbHLH96 under drought stress (Fig. 10). Briefly, drought stress induces *SlbHLH96* expression. SlbHLH96 directly binds to *cis*-elements in the *SlCYP707A2* promoter and downregulates its transcription, leading to increased levels of endogenous ABA, which, in turn, regulates the expression of ABA response-related genes. Furthermore, SlbHLH96 physically interacts with SlERF4, and the SlbHLH96–SlERF4 complex controls the expression of genes encoding antioxidants and stress-related genes. The study unveils novel mechanisms by which SlbHLH96 confers drought tolerance to tomato plants, thus providing important clues for breeding drought-resistant crops.

## Materials and methods

### Plant growth conditions

The tomato cultivar ‘Alisa Craig’ (AC) was used in this study and it also served as the transgene recipient. The plants were cultivated in growth chambers under a 16-h day (at 25°C), 8-h night (at 22°C) cycle and 80% relative humidity.

### Abiotic stress and hormone treatments for gene expression analysis

Surface-sterilized AC seeds were planted on ½ MS (Murashige–Skoog) medium plates for germination. Seedlings of identical size were moved to ½ MS medium plates after 7 days. For treatment with cold stress, the medium plates were transferred to an illuminating incubator at 4°C and sampled at 0, 1, 3, 6, 12, and 24 h. For heat treatment, the plates were placed in an illuminating incubator at 42°C and sampled at 0, 2, 4, 6, 12, and 24 h. For NaCl treatment, seedlings of similar size were transferred to ½ MS medium plates supplemented with 200 mM NaCl and incubated for 0, 1, 3, 6, 12, or 24 h for sampling. For PEG treatment, seedlings of similar size were transferred to ½ MS medium plates infused with different concentrations of PEG (average molecular weight 8000) solutions to achieve low water potentials from −0.25 to −1.7 MPa and incubated for 12 h. To detect the expression of *SlbHLH96* in response to exogenous hormones, treatments were performed as follows: 7-day-old seedlings with similar size grown on ½ MS medium were transferred to ½ MS medium plates supplemented with 0, 10 μM ABA, 10 μM IAA (indole-3-acetic acid), 10 μM GA_3_ (gibberellic acid 3), 10 μM SA (salicylic acid), or 10 μM JA (jasmonic acid), and incubated for 0, 1, 3, 6, 12, and 24 h.

### Subcellular localization of SlbHLH96

The full-length complete coding sequence (CDS) of *SlbHLH96* without the stop codon was constructed into a 35S promoter-driven pCAMBIA2300–GFP vector, resulting in the 35S-SlbHLH96–GFP plasmid. Leaves of *Nicotiana benthamiana* plants were infiltrated with the *Agrobacterium* strain GV3101 harboring the 35S-SlbHLH96–GFP plasmid or the empty vector of pCAMBIA2300–GFP (35S-GFP; as a control). Co-transformation of a red fluorescent protein (RFP) coupled with the nucleus marker mCherry made it possible to observe nuclei. After 48 hours, the fluorescence signals from the GFP protein expressed in the epidermal cells were observed with a BX53 (Olympus, Japan).

### Transcriptional activation analysis in yeast

The CDS and truncation of *SlbHLH96* were inserted into the pGBKT7 vector. The plasmids were inserted into Y2H-Gold, and were then grown on SD/−Leu and SD/−Leu/−Trp/−His medium at 30°C for 3 days.

### Tomato transformation

The full-length CDS of *SlbHLH96* was amplified by PCR from the first-strand tomato cDNA synthesized with the *SlbHLH96*-specific primer. Then, the *SlbHLH96* CDS was constructed into the plant expression vector pBI121. Finally, the recombinant vector was introduced into tomato cultivar AC by tissue culture-based *Agrobacterium*-mediated stable transformation (strain GV3101).

### RNA extraction and qRT–PCR analysis

Total RNA was isolated from AC tomato leaves using TRIzol (Tiangen, China). The cDNA was synthesized from the total RNA using the M-MLV Reverse Transcriptase kit (Vazyme, China). qRT–PCR reactions were performed with Tip Green SuperMix (TransGen, China). The relative expression was calculated using the 2^−ΔΔCt^ method. The *SlACTIN7* gene was used as a reference gene. Primer sequences in this study are listed in Supplementary Data Table 1.

### Methods for physiological measurements

NBT and DAB staining assays were performed as previously described [73]. O_2_^⋅−^ content and H_2_O_2_ content were determined using Solarbio detection kits (Solarbio, China).

The relative electrolytic leakage was measured to assess injuries to biological membranes as described previously [74]. The MDA content and proline content were measured as previously described [74, 75]. The activities of SOD and POD were measured as previously described [74].

### Measurement of ABA content

Endogenous ABA was extracted from freshly collected tomato leaves using extraction buffer [methanol:isopropanol:acetic acid = 20:79:1 (v:v:v)]. ABA content was determined using a UPLC–MS/MS system (QTRAP™ 5500 LC/MS/MS, USA).

### Virus-induced gene silencing

VIGS assays were conducted as previously described [76, 77]. A particular 300-bp sequence from *SlERF4* or *SlbHLH96* was designed using the SGN VIGS Tool (http://vigs.solgenomics.net/). A fragment of *SlERF4* or *SlbHLH96* was inserted into the pTRV2 vector for the construction of recombinant plasmid pTRV2:*SlERF4* and pTRV2:*SlbHLH96*, respectively. pTRV2:*00* (negative control), pTRV2-*SlPDS* (positive control), pTRV2:*SlERF4*, or pTRV2:*SlbHLH96* was mixed at a 1:1 ratio with pTRV1. The cotyledons of tomato plants were infiltrated with inoculant of *Agrobacterium* suspensions (OD_600_ = 1.0). When pTRV2-*SlPDS* plants showed a photo-bleached phenomenon, the silencing efficiency in pTRV2:*SlERF4* or pTRV2:*SlbHLH96* plants was analyzed using qRT–PCR.

### Bimolecular fluorescence complementation assay

The full-length CDS of *SlbHLH96* was cloned into pSPYCE vector to fuse with half of a YFP protein (SlbHLH96–cYFP). The full-length CDS of *SlERF4* was cloned into a pSPYNE vector to fuse with half of a YFP protein (SlERF4–nYFP). The recombinant plasmids were transformed into GV3101, which were then used to co-infiltrate *N. benthamiana* leaves. After 48 hours, fluorescence was observed with the BX53 (Olympus, Japan).

### Yeast two-hybrid assay

The full-length *SlERF4* and truncation of *SlbHLH96* were introduced into the pGADT7 and pGBKT7 vectors, respectively. The plasmids were introduced into yeast strain AH109 and grown on −Leu/−Trp/−His/−Ade medium (Coolaber, China).

### GST pull-down

Full-length *SlbHLH96* and *SlERF4* were inserted into the pMAL-c5X and pET42a vectors, respectively. The fusion proteins were purified with Amylose resin (NEB, USA) and Glutathione resin (GenScript, China), respectively. The GST pull-down assays were performed according to the MagneGST™ protein purification system User Manual (Promega, USA). The proteins were detected by western blotting with anti-MBP antibody and anti-GST antibody.

### Split-luciferase assay

Full-length *SlbHLH96* and *SlERF4* were cloned into the pCAMBIA1300–cLuc and pCAMBIA1300–nLuc vectors, respectively. The recombinant plasmids were transformed into *Agrobacterium tumefaciens* strain GV3101, and were then used to co-infiltrate *N. benthamiana* leaves. After 3 days, fluorescence was detected by a camera system (Lumazone Pylon 2048B, Princeton, USA).

### Dual-luciferase assay

The CDS of *SlbHLH96* and *SlERF4* was cloned into the pGreen62-SK vector. The promoters of *SlCYP707A2*, *SlCAT1*, and *SlPOD* were introduced into the pGreen0800-LUC vector, respectively. The recombinant vectors were transformed into GV3101 (pSoup-19) and infiltrated into 4-week-old *N. benthamiana* leaves. The Dual-Luciferase^®^ kit (Promega, USA) was used for dual-luciferase assays.

### Yeast one-hybrid assay

The promoter regions (P1 with one G-box and one E-box; P2 with 2 E-boxes) of *SlCYP707A2* were inserted to pHis2 and transformed into the Y187 yeast strain. The recombined yeast strain was transformed with the SlbHLH96-pGADT7 plasmid and the empty pGADT7 plasmid, respectively. The interactions between SlbHLH96 and *SlCYP707A2* promoter regions were indicated by the growth of the colony on SD/−Leu/−Trp/−His in the presence of 3-AT.

### Electrophoretic mobility shift assay

The CDS of *SlbHLH96* was cloned into pMAL-c5X to fuse with MBP. The SlbHLH96-MBP fusion protein was induced in *Escherichia coli* BL21 (DE3). EMSA was conducted as previously described [78].

### Statistical analysis

Data are reported as the means ± standard deviation. Statistical significance was determined by one-way ANOVA (Tukey’s test) using SPSS (version 26.0, USA). Variations were considered significant if *P* < .05. In some cases, significant differences in mean values, determined by Student’s t-test, are indicated by asterisk(s) (^*^P < .05; ^**^P < .01).

## Acknowledgements

The authors thank Dr Qingmei Guan from Northwest A&F University and graduate students (Xuewei Li, Chundong Niu, Dehui Zhang, Lijuan Jiang, Xiaoxia Shen, Fang Zhi, Fuguo Cao, Junxing Guo, Zeyuan Liu, and Gege Qin) for providing experimental consultation. The authors are grateful to experimentalists Minrong Luo and Hua Zhao (Northwest A&F University) for providing professional technical assistance.

This work was funded by the National Natural Science Foundation of China (grant numbers 31671273 and 31701925), the Key Research and Development Plan of Shaanxi Province (grant number 2020ZDLNY01-03), and the 100 Talents Plan of Shaanxi Province.

## Author contributions

Y.(Yunfei)L., X.Z., and J.Z. conceived the experiments. Y.(Yunfei)L. and F.M. performed the experiments. B.L., C.G., T.H., M.Z., and Y.(Yan)L. participated in the production of the experiment materials. Y.(Yunfei)L. wrote the paper. Y.(Yunfei)L., X.Z., and J.Z. revised the paper.

## Data availability

The data that support the results are provided in this paper and its supplementary files.

## Conflict of interest

The authors declare no competing interests.

## Supplementary Data

Supplementary data is available at *Horticulture Research* online.

## Supplementary Material

supp_data_uhac198
