# Supplementary material for: A bHLH transcription factor, SlbHLH96, promotes drought tolerance in tomato
Source: Hortic Res. 2022 Sep 6;9:uhac198. doi: 10.1093/hr/uhac198 (PMC9714257; doi:10.1093/hr/uhac198)
Supplement: supp_data_uhac198 [file supp_data_uhac198.zip › SlbHLH96_Supplementary_8-22-2022 V3.docx]

A bHLH Transcription Factor SlbHLH96 Promotes Drought Tolerance in Tomato

**Supplementary information**

This file contains Supplementary Figures S1-S6 and Table S1.


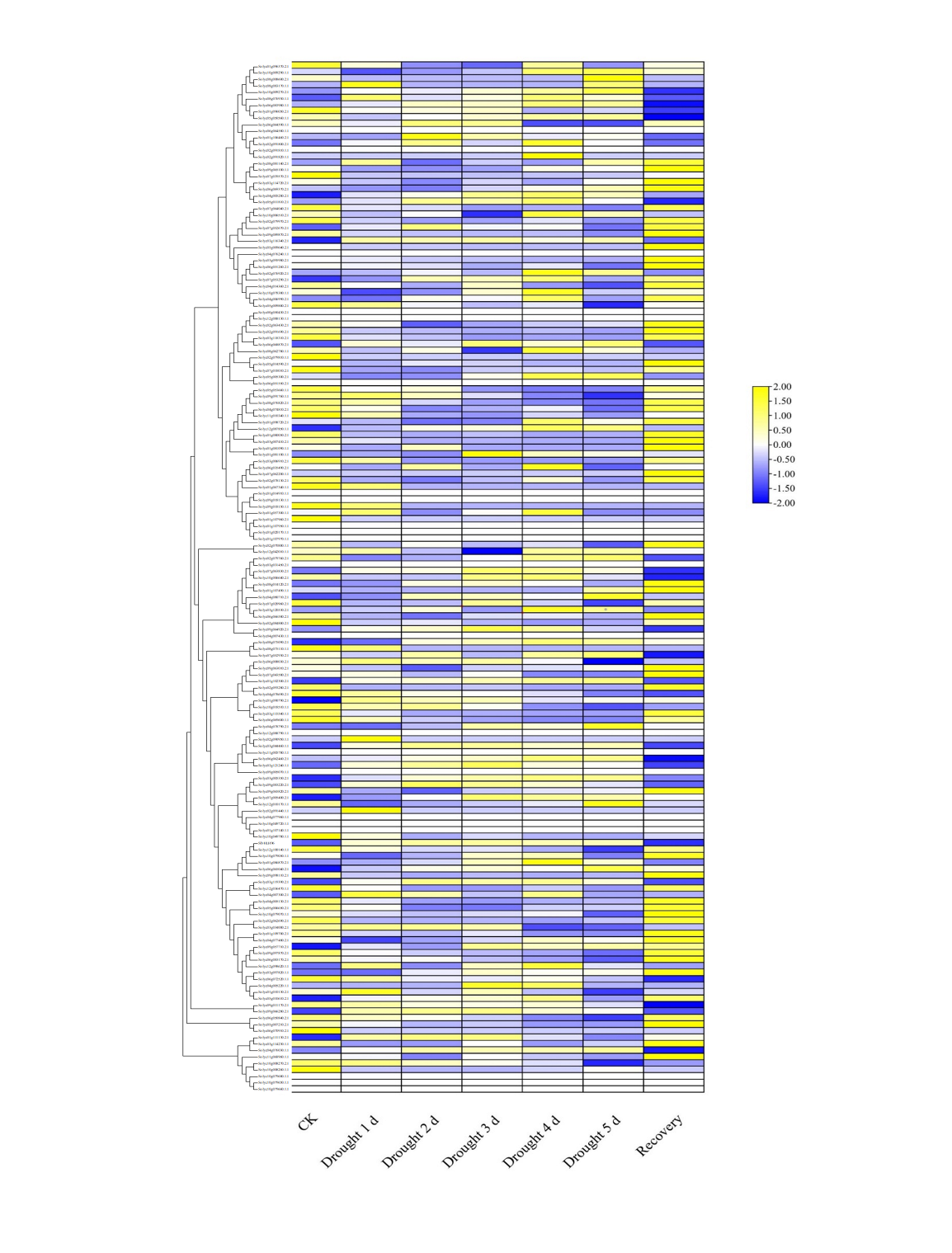


**Supplementary Fig. S1** **The expression profiles of *SlbHLH* genes in drought stress.** Heatmap of RNA-seq data. Four-week-old tomato AC plants grown in soil were withdrawn from water for 0, 1, 2, 3, 4, or 5 days (d) and then rewatered (Recovery).


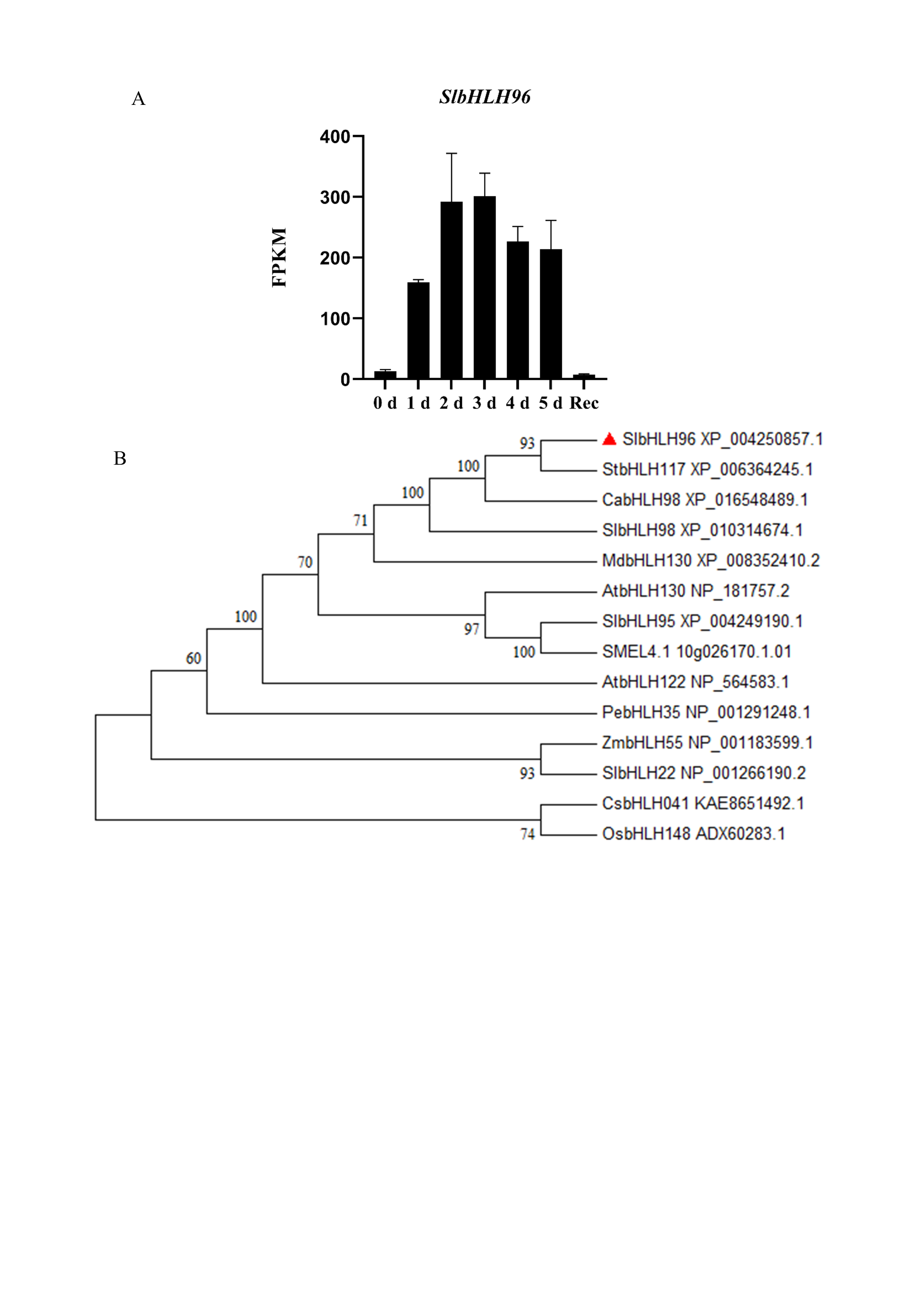


**Supplementary Fig. S2 Identification and phylogenetic analysis of SlbHLH96*.* A** RNA-seq data shown that *SlbHLH96* is upregulated by drought stress treatment in tomato. Four-week-old tomato AC plants grown in soil were withdrawn from water for 0, 1, 2, 3, 4, or 5 days (d) and then rewatered (Rec). **B** A phylogenetic analysis of SlbHLH96 and bHLH proteins in other plant species. Full-length of protein sequences were used to construct the phylogenetic consensus tree in MEGA7 using the neighbor-joining method (1000 replicates for computing the bootstrap values). Sl (*Solanum lycopersicum*), St (*Solanum tuberosum*), SMEL (*Solanum melongena*), Ca (*Capsicum annuum*), Md (*Malus*×*domestica*), At (*Arabidopsis thaliana*), Os (*Oryza sativa*), Zm (*Zea mays*), Pe (*Populus euphratica*), Cs (*Cucumis sativus*).


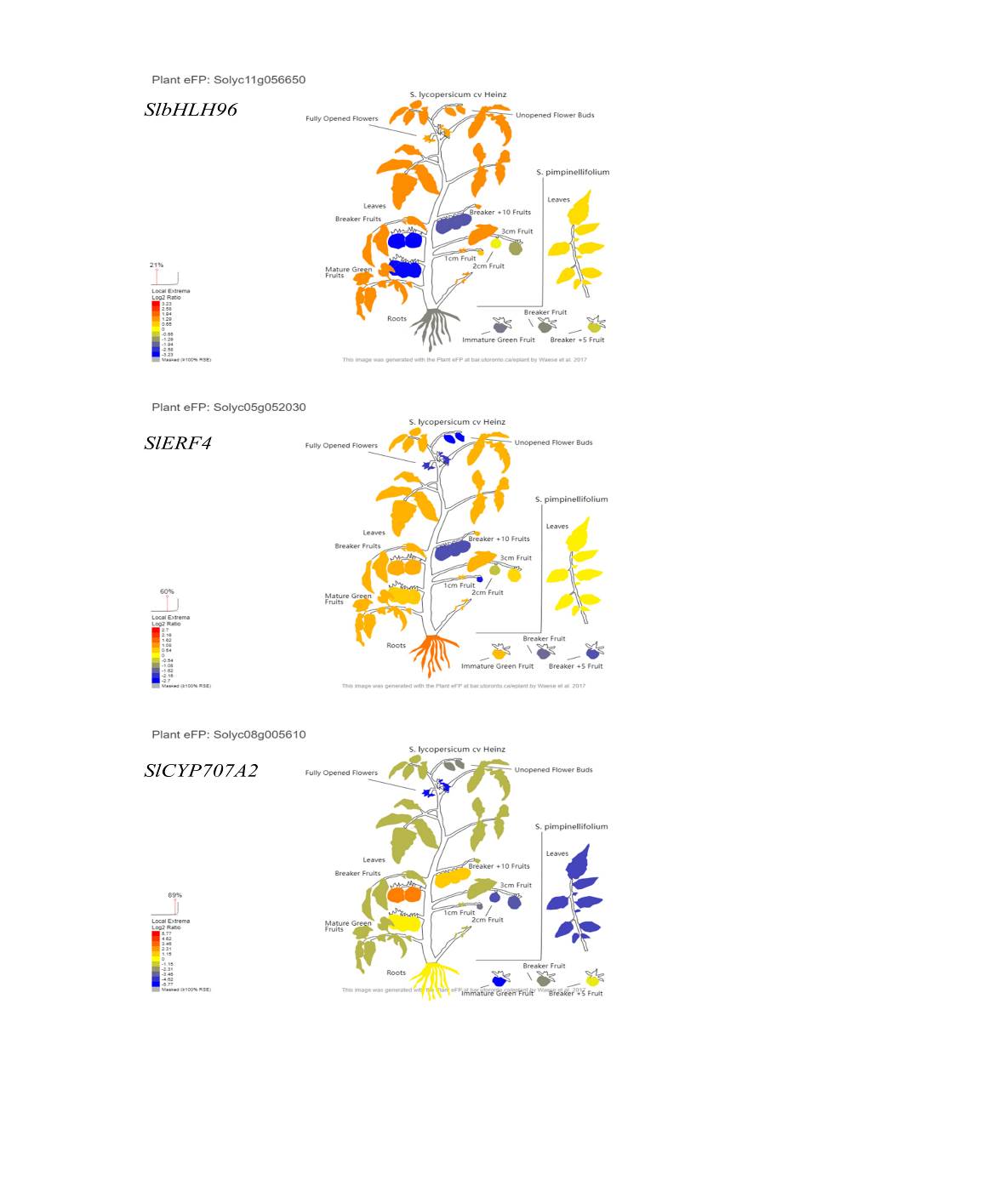


**Supplementary Fig. S3** **Tissue specific expression patterns of *SlbHLH96*, *SlERF4* and *SlCYP707A2****.* Data are Illumina-derived and RPKM-normalized. These images were generated with the Plant eFP at bar.utoronto.ca/eplant as described by by Waese et al. (2017).


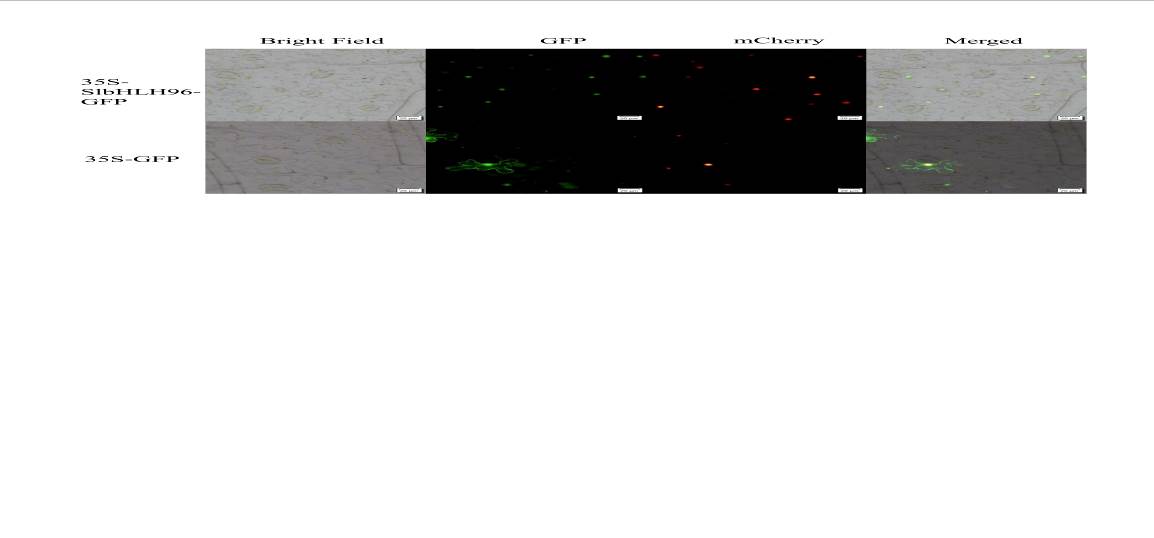


**Supplementary Fig. S4** **Subcellular localization of SlbHLH96**. SlbHLH96 was fused with a GFP for transient expression in 4-week-old tobacco leaves. mCherry stands for fluorescent signal from a red fluorescent protein (RFP) fused with a nucleus-localized marker mCherry. Bar = 20 μm.


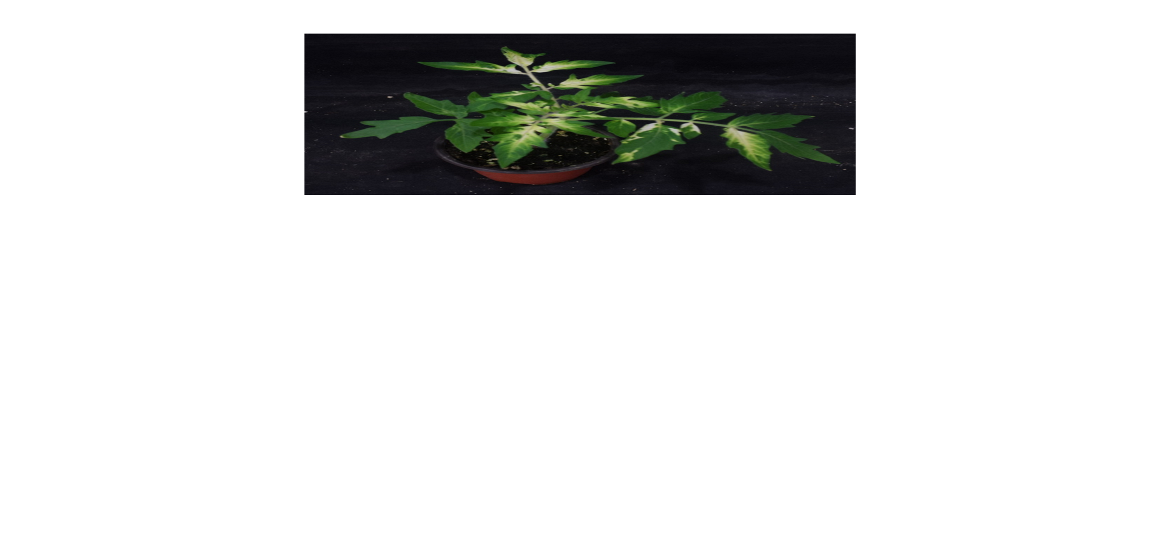


**Supplementary Fig. S5** **Phenotype of *SlPDS* silenced plant by VIGS.** Phenotype of *SlPDS* silenced plant, TRV1+TRV2-*SlPDS* (positive control)


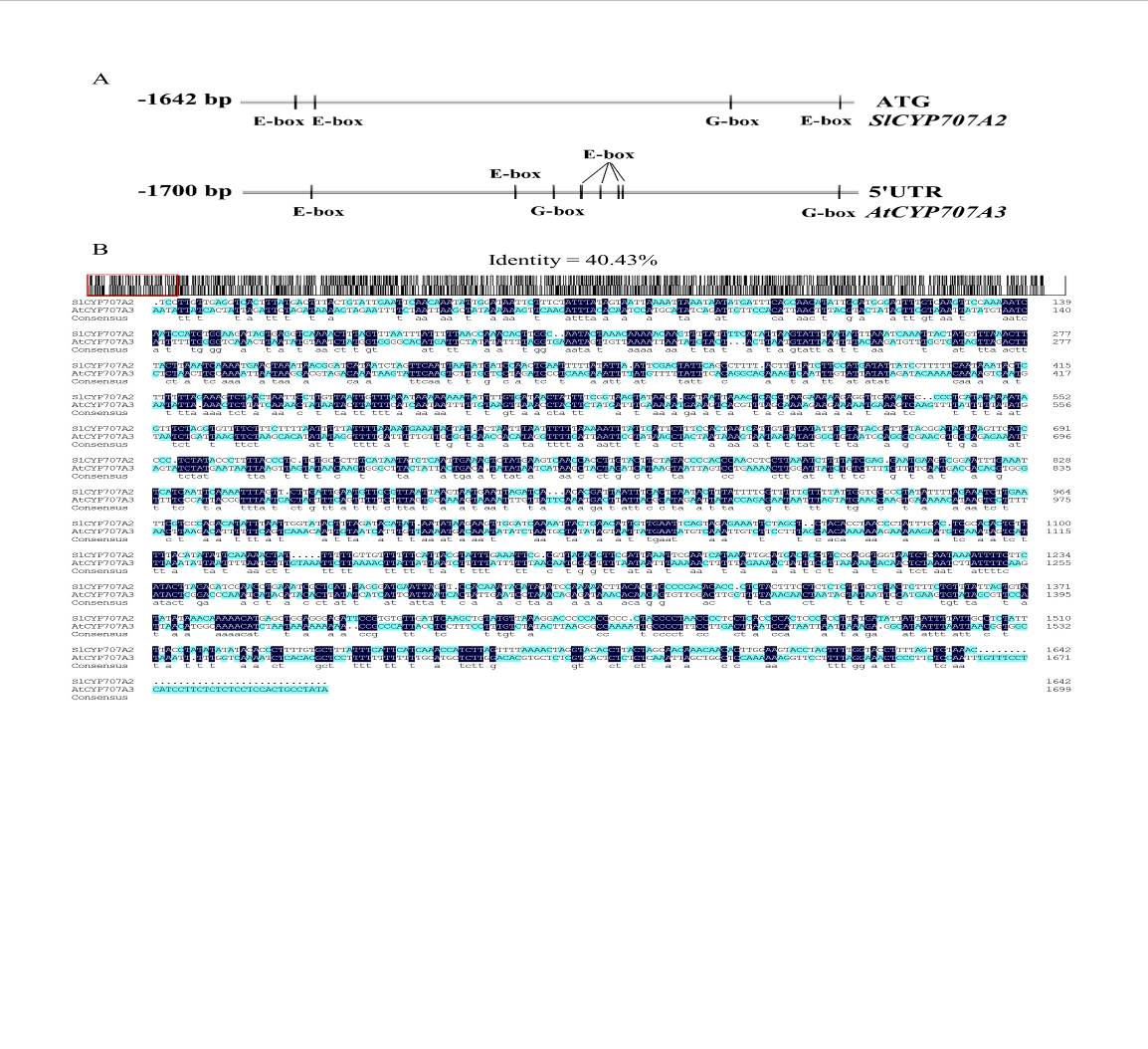


**Supplementary Fig. S6 Multiple sequence alignment of putative promoter regions of *SlCYP707A2* and *AtCYP707A3*. A** Schematic diagrams of G-box and E-box motifs in the promoter regions of *SlCYP707A2* and *AtCYP707A3*. **B** Multiple sequence alignment of *SlCYP707A2* and *AtCYP707A3* promoters.


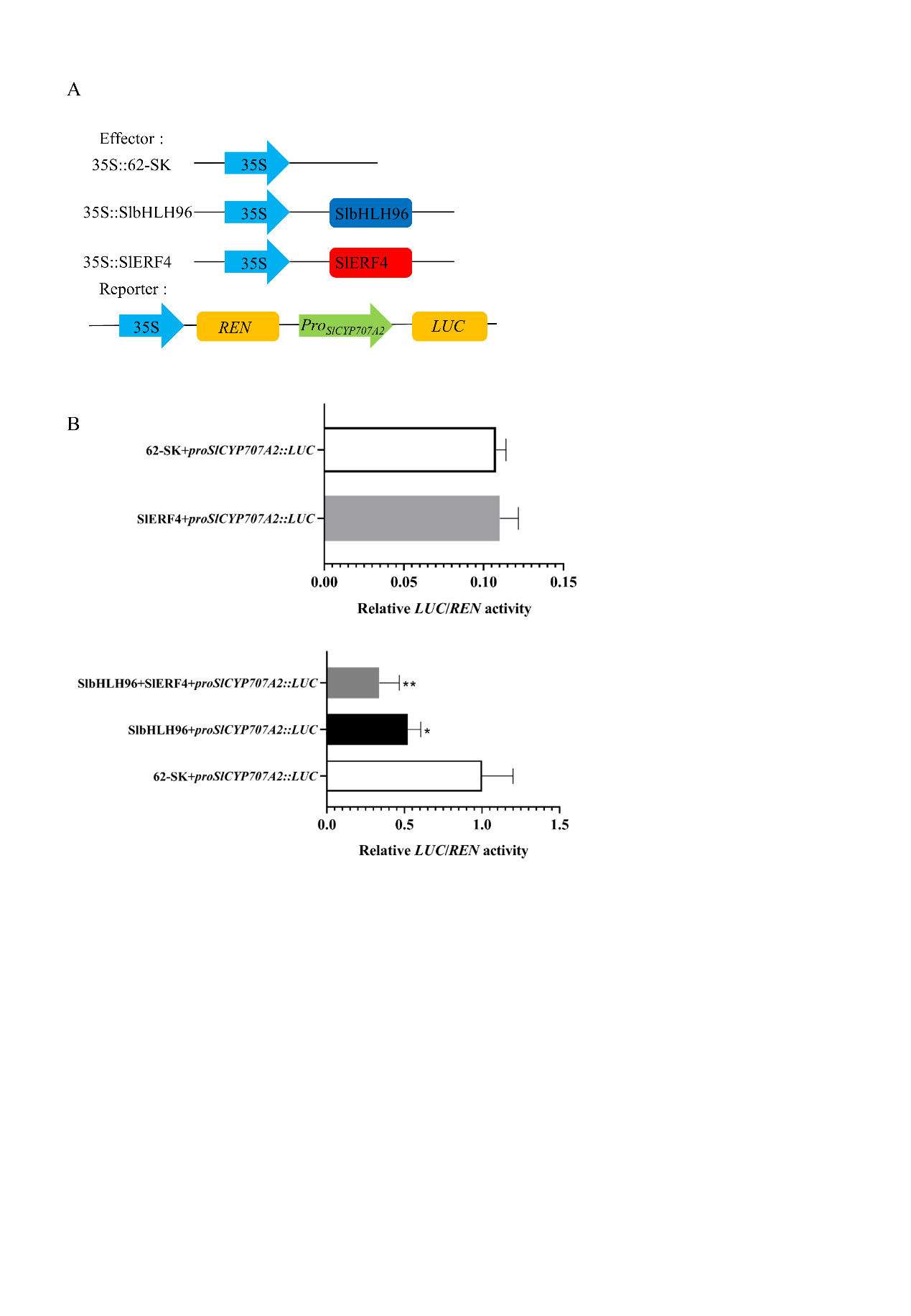


**Supplementary Fig. S7 The role of interaction between SlbHLH96 and SlERF4 in the regulation of the *SlCYP707A2*. A** Schematic representation of the reporter and effector. **B** Relative luciferase activity from the dual luciferase reporter assays in *N. benthamiana* leaves. The data are means ± SD (n = 3). Significant differences in mean values are indicated by asterisk(s) (* p < 0.05,** p < 0.01) determined by Student’s t-tests.


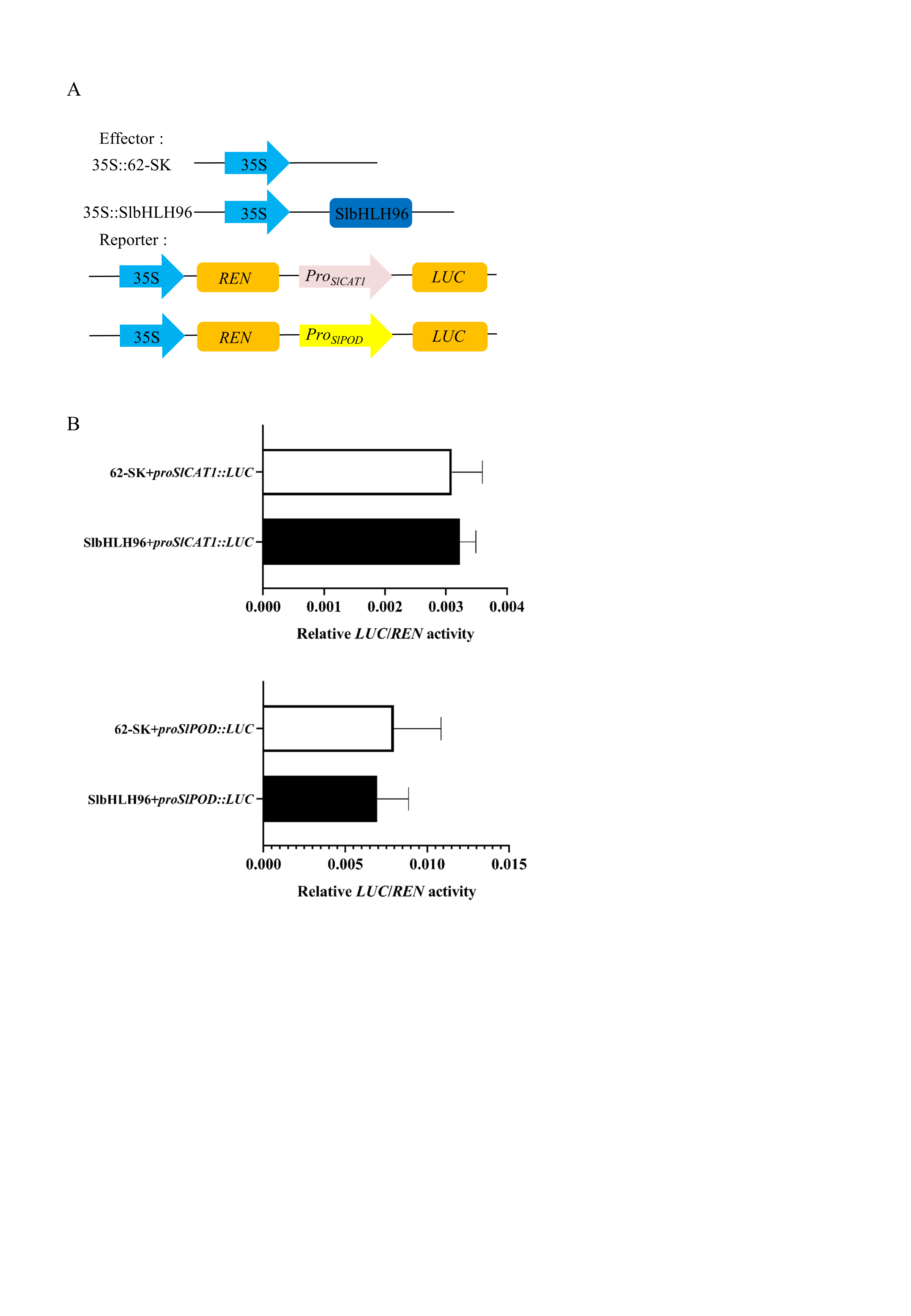


**Supplementary Fig. S8 SlbHLH96 could not regulate *SlCAT1 and SlPOD*.** **A** Schematic representation of the reporter and effector. **B** Relative luciferase activity from the dual luciferase reporter assays in *N. benthamiana* leaves. The data are means ± SD (n = 3). Significant differences in mean values are indicated by asterisk(s) (* p < 0.05) determined by Student’s t-tests.

| **Supplementary Table S1. Primers used in this study** | |  |
| --- | --- | --- |
| Name | Primer sequence (5′ to 3′) | Purpose |
| OE-SlbHLH96-F | TTGGAGAGGACACGCTCGAGGAAAAATGGATCATAGTCGTG | SlbHLH96 OE construct |
| OE-SlbHLH96-R | CATTAAAGCAGGACTCTAGACATCCTAACTTCACCTTTTGATC |  |
| GFP-SlbHLH96-F | ACACGGGGGACGAGCTCGGTACCATGGATCATAGTCGTGTTGAGCAG | SlbHLH96-GFP construct |
| GFP-SlbHLH96-R | CTCACCATGGTGTCGACTCTAGACTTATTTATGCACGTACATTTGGC |  |
| TRV2-SlbHLH96-F | AAGGTTACCGAATTCTCTAGAATGGATCATAGTCGTGTTGAGCAG | SlbHLH96 VIGS construct |
| TRV2-SlbHLH96-R | GAGACGCGTGAGCTCGGTACCTTTGTAGGAAAATTCCCAGAGTTAG |  |
| TRV2-SlERF4-F | AAGGTTACCGAATTCTCTAGACAAGATGAAGGATTAACATTAGAACTC | SlERF4 VIGS construct |
| TRV2-SlERF4-R | GAGACGCGTGAGCTCGGTACCGGAGCGGAGATTGAAGTAACGG |  |
| cYFP-SlbHLH96-F | GCTGTACAAGGGATCCATGGATCATAGTCGTGTTGAGCAG | SlbHLH96-cYFP construct |
| cYFP-SlbHLH96-R | TTCGAGCTCTATCCCGGGTTACTTATTTATGCACGTACATTTGGC |  |
| nYFP-SlERF4-F | CAACATCGAGGGATCCATGACGAAACAAGATGAAGGATTAAC | SlERF4-nYFP construct |
| nYFP-SlERF4-R | TTCGAGCTCTATCCCGGGCTACACCAACTCCATCTTGTTCTCTC |  |
| cLUC-SlbHLH96-F | GGGGACAAGTTTGTACAAAAAAGCAGGCTTCATGGATCATAGTCGTGTTGAGCAG | SlbHLH96-cLUC construct |
| cLUC-SlbHLH96-R | GGGGACCACTTTGTACAAGAAAGCTGGGTCTTACTTATTTATGCACGTACATTTGGC |  |
| nLUC-SlERF4-F | GGGGACAAGTTTGTACAAAAAAGCAGGCTTCATGACGAAACAAGATGAAGGATTAAC | SlERF4-nLUC construct |
| nLUC-SlERF4-R | GGGGACCACTTTGTACAAGAAAGCTGGGTCCACCAACTCCATCTTGTTCTCTC |  |
| GST-SlERF4-F | GGATATCGGGGATCCGAATTCATGACGAAACAAGATGAAGGATTAAC | SlERF4-GST  construct |
| GST-SlERF4-R | GAGTGCGGCCGCAAGCTTGTCGACCACCAACTCCATCTTGTTCTCTC |  |
| MBP-SlbHLH96-F | GAGGGAAGGATTTCACATATGATGGATCATAGTCGTGTTGAGCAG | SlbHLH96-MBP construct |
| MBP-SlbHLH96-R | CCTGCAGGGAATTCGGATCCTTACTTATTTATGCACGTACATTTGGC |  |
| AD-SlERF4-F | GTACCAGATTACGCTCATATGATGACGAAACAAGATGAAGGATTAAC | SlERF4-pGADT7 construct |
| AD-SlERF4-R | ATGCCCACCCGGGTGGAATTCCTACACCAACTCCATCTTGTTCTCTC |  |
| BK-SlbHLH96-F | TCTCAGAGGAGGACCTGCATATGATGGATCATAGTCGTGTTGAGCAG | SlbHLH96-pGBKT7 construct |
| BK-SlbHLH96-R | GGTCGACGGATCCCCGGGAATTCTTACTTATTTATGCACGTACATTTGGC |  |
| BK-SlbHLH96-N-F | TCTCAGAGGAGGACCTGCATATGATGGATCATAGTCGTGTTGAGCAG | SlbHLH96-N-pGBKT7 construct |
| BK-SlbHLH96-N-R | GGTCGACGGATCCCCGGGAATTCTTACCTCTTTGCCCTGACATTCAAGTG |  |
| BK-SlbHLH96-C-F | TCTCAGAGGAGGACCTGCATATGGGTTGTGCCACTCACCCTCGTAGC | SlbHLH96-C-pGBKT7 construct |
| BK-SlbHLH96-C-R | GGTCGACGGATCCCCGGGAATTCTTACTTATTTATGCACGTACATTTGGCG |  |
| BK-SlbHLH96-CΔ1-F | TCTCAGAGGAGGACCTGCATATGTGGGAAGATTCACACATTTTGTCC | SlbHLH96-CΔ1-pGBKT7 construct |
| BK-SlbHLH96-CΔ2-F | TCTCAGAGGAGGACCTGCATATGCTTACCCGTTATAATAGTTCGCCTGC | SlbHLH96-CΔ2-pGBKT7 construct |
| BK-SlbHLH96-CΔ3-F | TCTCAGAGGAGGACCTGCATATGAGATCGGATTTTGTTGTACCAAAACAAG | SlbHLH96-CΔ3-pGBKT7 construct |
| 800LUC-SlCYP707A2-F | CACTATAGGGCGAATTGGGTACCGAGGTCACTTTATGACTTTACTGTATTG | SlCYP707A2-800LUC construct |
| 800LUC-SlCYP707A2-R | TATGTTTTTGGCGTCTTCCATGGGAACTAAAAGGTACCAAAAGTAGG |  |
| 62SK-SlbHLH96-F | AGAGGACAGCCCAAGCTGAGCTCATGGATCATAGTCGTGTTGAGCAG | SlbHLH96-62SK construct |
| 62SK-SlbHLH96-R | TTTCAGCGTACCGAATTGGTACCTTACTTATTTATGCACGTACATTTGGC |  |
| pHis-SlCYP707A2P1-F | GACTCACTATAGGGCGAATTCCATATATCCAAAAACTTACACGTGC | SlCYP707A2-P1-pHIS construct |
| pHis-SlCYP707A2P1-R | GATTCGCGAACGCGTGAGCTCGAACTAAAAGGTACCAAAAGTAGG |  |
| pHis-SlCYP707A2P2-F | GACTCACTATAGGGCGAATTCGAGGTCACTTTATGACTTTACTGTATTG | SlCYP707A2-P2-pHIS construct |
| pHis-SlCYP707A2P2-R | GATTCGCGAACGCGTGAGCTCGAACTAGATTATGATCCGTTATTTAG |  |
| AD-SlbHLH96-F | GTACCAGATTACGCTCATATGATGGATCATAGTCGTGTTGAGCAG | SlbHLH96-pGADT7 construct |
| AD-SlbHLH96-R | ATGCCCACCCGGGTGGAATTCTTACTTATTTATGCACGTACATTTGGC |  |
| EMSA-SlCYP707A2-1R | CTTCCACATGGATTGATTTTTGG | SlCYP707A2-EMSA probe construct |
| EMSA-SlCYP707A2-2F | CCAAACACTTGGCAATACTAAAC |  |
| EMSA-SlCYP707A2-3R | CACGGAATCTCCCTCCAGCTCATG |  |
| EMSA-SlCYP707A2-4F | CTTTATTTCATTCATCAAACCATC |  |
| EMSA-M13F | TGTAAAACGACGGCCAGT |  |
| EMSA-pHIS-R | GCCAGGAATTTCTAGACCGC |  |
| EMSA-SlCYP707A2-1Rmut | CTTCttttttGATTGATTTTTGGAACTTG | SlCYP707A2-EMSA mutant probe construct |
| EMSA-SlCYP707A2-2Fmut | CCAAAaaaaaaGCAATACTAAACAAAAAC |  |
| EMSA-SlCYP707A2-3Rmut | GGGGttttttTAAGTTTTTGGATATATG |  |
| EMSA-SlCYP707A2-4Fmut | CTAGGTACACCTTACTAGCAACAAACAAaaaaaaGAAGTACCTACTTTTGG |  |
| qPCR-SlACTIN7-F | AGGCAGGATTTGCTGGTGATGATGCT | qRT-PCR |
| qPCR-SlACTIN7-R | ATACGCATCCTTCTGTCCCATTCCGA |  |
| qPCR-SlbHLH96-F | CTGCTGCAGATACTTCATTGTC | qRT-PCR |
| qPCR-SlbHLH96-R | AATCTGTAATGCAATGCTCGTC |  |
| qPCR-SlERF4-F | CGGAGATAAGAGATCCAAGTCG | qRT-PCR |
| qPCR-SlERF4-R | CTTAAACGCTGCACAATCATAAGC |  |
| qPCR-SlCYP707A2-F | TCGAAAAAGGATACAATTCGATGCC | qRT-PCR |
| qPCR-SlCYP707A2-R | CTGCAATTTGTTCGTCAGTGAGTCC |  |
| qPCR-SlNCED1-F | AGGCAACAGTGAAACTTCCATCAAG | qRT-PCR |
| qPCR-SlNCED1-R | TCCATTAAAGAGGATATTACCGGGGAC |  |
| qPCR-SlPYL7-F | TCGGTGGGGACCACAAGTTAGC | qRT-PCR |
| qPCR-SlPYL7-R | CGATTGAAGGTTGCATTTCACGAT |  |
| qPCR-SlSnRK2.6-F | CAGCAATGACTTTGCTGTTTATGTCTG | qRT-PCR |
| qPCR-SlSnRK2.6-R | CGTGAATCCGAATTAGACGGATACC |  |
| qPCR-SlPP2C1-F | AGAATAAAGAAACCGAAACGAACGC | qRT-PCR |
| qPCR-SlPP2C1-R | ATCCTGAAGAAACGACGGGTAGATC |  |
| qPCR-SlPP2C4-F | GGTACTTTGAGTAGGGAAAGGGGTGA | qRT-PCR |
| qPCR-SlPP2C4-R | GAAATACGAGGATGGTTTAGTGCGTTA |  |
| qPCR-SlDREB1-F | GACTCATTGCCTCGCCCA | qRT-PCR |
| qPCR-SlDREB1-R | TTTCACCCAGTTCCTCCG |  |
| qPCR-SlDREB2A-F | CAATGAAGGGAAAGGGAAGC | qRT-PCR |
| qPCR-SlDREB2A-R | TACCCAACCATAACCTACTACC |  |
| qPCR-SlAREB1-F | TCCTTATGTGTTTAATGGTGGTTT | qRT-PCR |
| qPCR-SlAREB1-R | CATCGTTTTCTTCTTTTAGTTTCG |  |
| qPCR-SlSOD-F | ACATACAAAAATGGTGAAGGCC | qRT-PCR |
| qPCR-SlSOD-R | AGGATTGTAATGTGGTCCTGTT |  |
| qPCR-SlAPX1-F | CTGGTGTTGTTGCTGTTGAAG | qRT-PCR |
| qPCR-SlAPX1-R | GCTCTGGCTTGTCCTCTCTG |  |
| qPCR-SlCAT1-F | GGTGGATTATTTGCCCTCG | qRT-PCR |
| qPCR-SlCAT1-R | ACCTCTCCCCTGCCTGTTT |  |
| qPCR-SlPOD-F | GGTCAACGGATAGCATTGTTAC | qRT-PCR |
| qPCR-SlPOD-R | TTTATAGCGCCACAAACAGTTC |  |
| qPCR-SlAPX2-F | GGCTGGTGTTGTTGCTGTTG | qRT-PCR |
| qPCR-SlAPX2-R | TCAGGCAAGCGACCTTCAAC |  |
| qPCR-SlCAT2-F | AACAACTTCCCCGTCTTCTTC | qRT-PCR |
| qPCR-SlCAT2-R | TTAGGATTTGGCTTCAGAGCA |  |
